# Comparing Hydrolysis and Transglycosylation Reactions Catalyzed by *Thermus thermophilus* β-Glycosidase. A Combined MD and QM/MM Study

**DOI:** 10.3389/fchem.2019.00200

**Published:** 2019-04-10

**Authors:** Sonia Romero-Téllez, José M. Lluch, Àngels González-Lafont, Laura Masgrau

**Affiliations:** ^1^Departament de Química, Universitat Autònoma de Barcelona, Barcelona, Spain; ^2^Institut de Biotecnologia i de Biomedicina, Universitat Autònoma de Barcelona, Barcelona, Spain

**Keywords:** glycosylhydrolase, transglycosylation, QM/MM, glycans, hydrolysis, glycosyl-enzyme complex, GH1

## Abstract

The synthesis of oligosaccharides and other carbohydrate derivatives is of relevance for the advancement of glycosciences both at the fundamental and applied level. For many years, glycosyl hydrolases (GHs) have been explored to catalyze the synthesis of glycosidic bonds. In particular, retaining GHs can catalyze a transglycosylation (T) reaction that competes with hydrolysis (H). This has been done either employing controlled conditions in wild type GHs or by engineering new mutants. The goal, which is to increase the T/H ratio, has been achieved with moderate success in several cases despite the fact that the molecular basis for T/H modulation are unclear. Here we have used QM(DFT)/MM calculations to compare the glycosylation, hydrolysis and transglycosylation steps catalyzed by wild type *Thermus thermophilus* β-glycosidase (family GH1), a retaining glycosyl hydrolase for which a transglycosylation yield of 36% has been determined experimentally. The three transition states have a strong oxocarbenium character and ring conformations between ^4^H_3_ and ^4^E. The atomic charges at the transition states for hydrolysis and transglycosylation are very similar, except for the more negative charge of the oxygen atom of water when compared to that of the acceptor Glc. The glycosylation transition state has a stronger S_*N*_2 character than the deglycosylation ones and the proton transfer is less advanced. At the QM(PBE0/TZVP)/MM level, the TS for transglycosylation has shorter O4_GLC_-C1_FUC_ (forming bond) distance and longer OE2_GLU338_-C1_FUC_ (breaking) distance than the hydrolysis one, although the H_ACC_ proton is closer to the Glu164 base in the hydrolysis TS. The QM(SCC-DFTB)/MM free energy maxima show the inverted situation, although the hydrolysis TS presents significant structural fluctuations. The 3-OH_GLC_ group of the acceptor Glc (transglycosylation) and WAT432 (neighbor water in hydrolysis) are identified to stabilize the oxocarbenium transition states through interaction with O5_FUC_ and O4_FUC_. The analysis of interaction suggests that perturbing the Glu392-Fuc interaction could increase the T/H ratio, either by direct mutation of this residue or indirectly as reported experimentally in the Asn390I and Phe401S cases. The molecular understanding of similarities and differences between hydrolysis and transglycosylation steps may be of help in the design of new biocatalysts for glycan synthesis.

## Introduction

Carbohydrates and their derivatives (glycoconjugates and glycosides) play important biological functions, including energy storage, structural roles and also the encoding of a molecular and cell recognition language that drives the specificity of such processes. Accordingly, glycans are vital for normal life development and are also key factors in the progression of many diseases (from pathogen infection to cancer) (Seeberger and Cummings, 2017). In order to develop fundamental and applied research in the glycosciences field, access to pure glycans and in sufficient amounts is needed. Glycans biosynthesis involves the action of a repertoire of enzymes, amongst them glycosyltransferases (GTs, which catalyze the synthesis of a new glycosidic bond) and glycoside hydrolases (GHs). These enzymes catalyze the corresponding reaction with two possible stereochemical outcomes, that is, retention or inversion of the configuration at the anomeric carbon of the transferred/hydrolyzed sugar.

Besides being the target of drug design investigations, carbohydrate-active enzymes are of interest in glycans processing and synthesis, an important area of fundamental research and also for the preparation of commercially-valuable products. Glycoside hydrolytic enzymes (e.g., amylases, cellulases) are used in the e.g., food, textile, detergents, cosmetics, pulp, and paper industries, representing around one third of the global industrial enzyme market (Plou et al., 2007; Sajith et al., 2016). The enzymatic synthesis of glycosidic bonds by carbohydrate-active enzymes has also been a subject of study for over 60 years and different strategies have been developed that circumvent or complement chemical approaches, which usually require multiple protection, and deprotection steps to obtain the desired oligosaccharide (Kiessling and Splain, 2010; Bissaro et al., 2015b; Danby and Withers, 2016). In Nature, GTs are the main catalysts for the synthesis of glycosidic bonds. However, their broad application is hindered by the difficulties in their expression and purification and due to the economic costs of obtaining the nucleotide-phosphate sugars they use as donor substrates, despite progresses have been made in that direction (Field, 2011). As an alternative, GHs have been explored to catalyze glycosidic bond formation (Planas and Faijes, 2002; Cobucci-Ponzano et al., 2011; Bissaro et al., 2015b). GHs are more abundant than GTs, are much easier to obtain, cover a wide range of substrate specificities and their substrates are cheap.

**Figure 1:**
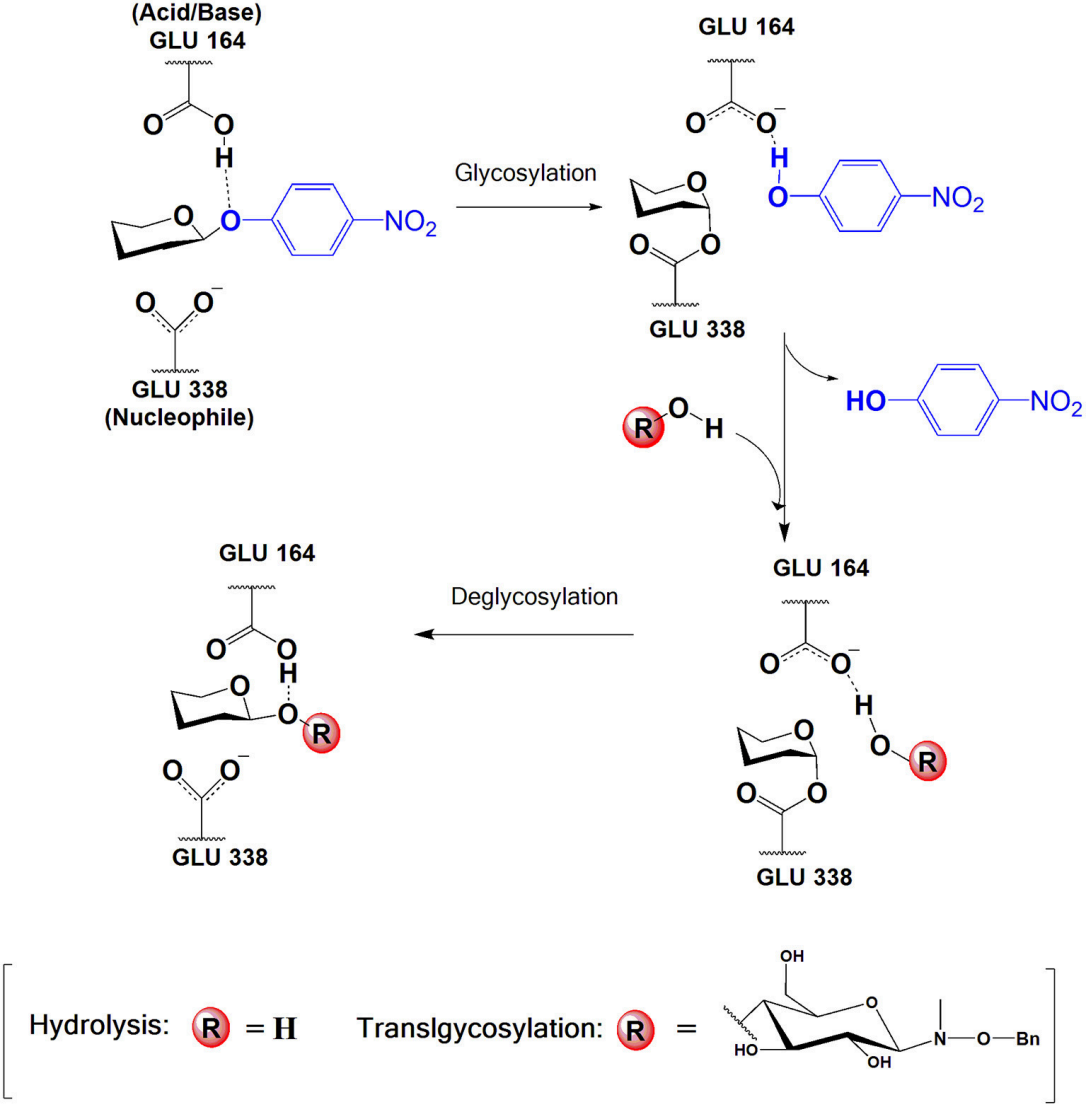
Catalytic mechanism of the glycosylation and deglycosylation (hydrolysis or transglycosylation) steps in retaining β-glycoside hydrolases. In this work, nucleophile and acid/base residues are GLU 338 and GLU 164, respectively, the donor substrate is *p*NP-Fuc and the acceptor substrate for transglycosylation is BnON(Me)-Glc.

In particular, retaining GHs can operate in a synthetic mode if the equilibrium is displaced toward glycoside bond formation (thermodynamically controlled processes) or by using activated glycosyl donors (kinetically controlled transglycosylation) (Planas and Faijes, 2002). Retaining GHs follow a double displacement mechanism in two subsequent steps with formation of a covalent glycosyl-enzyme intermediate. Two catalytic carboxyl groups (separated by ~5Å) act as general acid/base and nucleophile in the reaction (as exemplified in Figure 1 for the process studied in this work). In a first step (referred as glycosylation, G), the nucleophile attacks the anomeric carbon while the carboxylic acid protonates the glycosidic oxygen of the leaving group, and the glycosyl-enzyme intermediate is formed. In the final step (deglycosylation, D), the residue previously acting as an acid now deprotonates the incoming acceptor substrate that attacks the anomeric carbon forming the final product with net retention of the configuration. If the acceptor is a water molecule, hydrolysis (H) occurs. However, with the presence of a different suitable sugar acceptor, many retaining GHs are capable of catalyzing transglycosylation (T).

Different approaches and experimental conditions have been used to enhance the transglycosylation vs. hydrolysis (T/H) ratio, e.g., high acceptor concentrations, the use of highly reactive glycosyl donors (activated donors) like aryl glycosides or glycosyl fluorides, removal of the transglycosylation product or enzyme immobilization. With all, yields and regiospecificities are still low (Planas and Faijes, 2002). Engineering of GHs has also been investigated. A major breakthrough in the field was accomplished with the development of glycosynthases, in which hydrolysis is abolished by replacing the catalytic nucleophile by a non-nucleophilic residue and the use of activated substrates (Mackenzie et al., 1998; Malet and Planas, 1998; Moracci et al., 1998; Faijes and Planas, 2007; Cobucci-Ponzano and Moracci, 2012; Danby and Withers, 2016). Other studies have tried to understand the molecular determinants for having a transglycosylation vs. an hydrolytic enzyme activity by applying mutational studies (Bissaro et al., 2015b). Notably, Dion and coworkers (Feng et al., 2005) successfully applied directed evolution techniques to the β-glycosidase of *Thermus thermophilus* (Ttβ-gly), belonging to the CAZy family GH1 (Henrissat, 1991; Cantarel et al., 2009; Hart and Copeland, 2010), to increase its ability to synthesize oligosaccharides by transglycosylation.

Their findings were later “semi-rationalized” by realizing that mutations leading to an increase in the T/H ratio in Ttβ-gly were located at highly conserved positions at the−1 subsite (the one accommodating the hydrolyzed/transferred monosaccharide) (Teze et al., 2014). In this way, they created Ttβ-gly mutants (e.g., Tyr284Phe, Asn282Thr, Phe401Ser, Asn163Ala, Arg75Ala) that catalyzed transglycosylation between 4-nitrophenyl β-*D*-fucopyranoside (*p*NP-Fuc) (donor substrate) and *N*-methyl-*O*-benzyl-*N*-(β-*D*-glucopyranosyl)-hydroxylamine (BnON(Me)-Glc, acceptor), with increased yields of up to 82% against a 36% in wild type enzyme, although in all cases the catalytic efficiency is significantly reduced when compared to WT Ttβ-gly. The approach has since then been applied to other GHs families (glycosyl hydrolases have been classified into different families according to sequence similarity) (Henrissat, 1991; Cantarel et al., 2009; Teze et al., 2015; Saumonneau et al., 2016). The authors postulated that mutation of these well-conserved residues around the−1 subsite may induce lower stabilization of the deglycosylation transition states (TS), being the effect larger for hydrolysis than for transglycosylation reaction, therefore resulting in the higher T/H ratio measured experimentally (Teze et al., 2014). This suggests that the H and T transition states present different characteristics and stabilization interactions. For another family GH1 β-glucosidase (NkBgl), mutation of the catalytic acid/base glutamate to aspartate was also found to generate new transglycosylation products not produced by the wild-type enzyme (Jeng et al., 2012). The authors also proposed that this catalytic residue is important in substrate entry and product release and noticed the importance of aromatic rings in the aglycone site to facilitate acceptor (other than water) binding. Compiling evidences support the idea that the properties of the deglycosylation transition state, substrate-specific interactions involving the acceptor substrate and specific features to channel and retain water molecules close to the catalytic center, are key determinants for the T/H partition (Kempton and Withers, 1992; Bissaro et al., 2015a,b; David et al., 2017). Still, the molecular details for the basis of T/H modulation are unclear.

Computational studies on retaining GHs have mainly been focused on the glycosylation step, fewer to the hydrolysis one and very few to transglycosylation. For enzymes belonging to CAZy family GH1, the one investigated in this work, quantum mechanical molecular mechanical (QM/MM) studies on glycosylation and hydrolysis steps catalyzed by *Oryza sativa* (rice) β-glucosidase (Osβ-gly) acting on glucose disaccharides have been reported (Wang et al., 2011, 2013; Badieyan et al., 2012). They corroborated the predicted oxocarbenium-like nature of both TSs, which present the corresponding breaking bond already broken and the forming one far from established. The interaction of the C2 hydroxyl group from the sugar with the catalytic residues was also confirmed to play an important role in the catalytic reaction by facilitating the sugar ring conformational change toward the TS and by contributing to TS stabilization. This is in agreement with experimental data that have for long identified this interaction as important for TS stabilization, with a contribution of up to 10 kcal/mol (Zechel and Withers, 2000). Recently, the effect of such interaction on lowering the free energy barrier for the glycosylation and transglycosylation reactions catalyzed by a transglycosidase of CAZy family 72, was calculated to be of 11 and 16 kcal/mol, respectively (Raich et al., 2016). The effect of using different QM/MM partitions on the barrier heights of glycosylation and hydrolysis in Osβ-gly has also been investigated (Badieyan et al., 2012). It was concluded that a Tyr residue equivalent to Ttβ-gly Tyr284 (thus interacting with the catalytic nucleophile and the sugar O5 atom), has an important contribution to the energy profile, being the effect more significant for deglycosylation than for glycosylation. Importantly, the study showed that this residue had to be included in the QM region to obtain reliable potential energy barriers and ring conformations. The effect of other residues from the−1 subsite was also analyzed.

Very few QM/MM studies have compared hydrolysis and transglycosylation reactions for retaining glycosidases and, to the best of our knowledge, none has focused on family GH1. BB1K:AMBER QM/MM calculations on a β-galactosidase belonging to family GH2 were used to study the catalytic mechanism of glycosylation, hydrolysis and transglycosylation reactions, the latter leading to different regioproducts (Bráa et al., 2010). All TSs were characterized as dissociative and with the proton still attached to the acidic residue, although, unfortunately, more detailed geometric information on the transglycosylation TS was not given. The 2-OH group H-bond with the nucleophile was also related to pyranosyl ring distortion and TS stabilization. The energy barriers for transglycosylation were found to be higher than for hydrolysis by ~2–4 kcal/mol, and the origin of the observed regioselectivity was found to be thermodynamic more than kinetic. Recently, the hydrolysis and transglycosylation reactions catalyzed by a family GH3 β-glucosidase have been studied by umbrella sampling calculations at the SCC-DFTB/CHARMM level (Geronimo et al., 2018). Both reactions were found to have similar free energy barriers (~18 kcal/mol) but quite different TSs; the predicted TS for hydrolysis also differed from the ones previously described. For hydrolysis, the TS had the glycosyl-water bond practically formed (1.50 ± 0.04 Å), resulting in a reduced ionic character of the sugar which, according to the Cremer-Pople polar coordinates (Cremer and Pople, 1975) that describe ring conformations, presented a ^4^C_1_ conformation. For transglycosylation, an earlier TS was predicted (with the new bond only partially formed, 1.9 ± 0.1 Å), with the transferred sugar more positively charged and in a ^4^H_3_ conformation. In both cases, proton transfer to the catalytic base occurred late, especially in the hydrolysis step, where after approaching the base catalyst at 1.6 Å.

Here we present a QM(DFT)/MM study of the glycosylation, hydrolysis and transglycosylation steps catalyzed by wild-type Ttβ-gly using *p*NP-Fuc as donor and BnON(Me)-Glc as acceptor. Ttβ-gly catalyzes the hydrolysis of β-*D*-galactoside, β-*D*-glucoside, and β-*D*-fucoside derivatives, showing the highest activity with the latter (Dion et al., 1999). As mentioned, in the presence of a suitable acceptor, it can also catalyze transglycosylation; BnON(Me)-Glc has been reported to produce a high transglycosylation yield in the native enzyme and a sole regioisomer product (*N*-methyl-*O*-benzyl-*N*-(β-*D*-fucopyranosyl(1 → 4)β-*D*-glucopyranosyl)-hydroxylamine) (Teze et al., 2013). Our main goal is to gain understanding and compare the molecular mechanisms underlying hydrolysis and transglycosylation. It has been proposed that the main driving force for increasing the T/H ratio must be the relative destabilization of the hydrolysis TS as compared to the transglycosylation one, which implies that differential interactions are involved in each case.

## Methodolgy

### System Setup

Initial coordinates for the wild-type Ttβ-gly were taken from the corresponding X-ray structure with PDB code 1UG6 (resolution 0.99 Å) (Lokanath et al., in press). For the incorporation of the substrates into the enzyme active site, the 1UG6 coordinates were overlaid with those of the Osβ-gly structure crystalized with non-hydrolyzed cellotetraose (CTT) substrate(PDB code: 3F5J, resolution 1.95 Å) (Chuenchor et al., 2011). In this way, we took advantage of the position of CTT and modified it with PyMol program^1^, by building the fucose moiety from the CTT glucose ring closer to the catalytic residues (Glu338 and Glu164 of the 1UG6 wild-type structure). The second ring of the CTT substrate was used to build up the (*p*-nitrophenol (*p*NP)) leaving group of the *p*NP-Fuc substrate. Two crystallographic water molecules from the wild-type structure that clashed to the added substrate were removed; the remaining of crystallographic waters were kept in the model. Missing hydrogen atoms were added to the model and the ionizable residues were protonated using Propka3.0 (Olsson et al., 2011) at pH = 7.0, except for Glu338 and Glu392 that were modeled as deprotonated, Glu164 as protonated and His119 was protonated at Nδ. All molecular dynamics (MD) simulations were done using the ff14SB (Maier et al., 2015) Amber force field for the protein; GLYCAM06j (Kirschner et al., 2008) and GAFF (Wang et al., 2004) atom types and parameters were employed for the sugar moieties and the *p*NP group, respectively. In addition, several missing parameters corresponding to a bond, and several intramolecular angles and dihedrals of the *p*NP-Fuc substrate were completed searching for the corresponding atom types translation in GAFF and GLYCAM06j force field databases (those parameters are given in Supplementary Scheme S1 and Supplementary Table S1). Three sodium ions were added to neutralize the system, which was finally solvated in a cubic box of preequilibrated TIP3P water molecules, with no <15 Å from the edge of the water box to the nearest protein atom. The resulting system contained 70,227 atoms.

### Molecular Dynamics (MD) Simulation Details

The solvated Ttβ-gly/*p*NP-Fuc model system was relaxed by performing energy minimization at the MM level with the steepest descent and conjugate gradient methods. The minimization protocol consisted in a three-step process, firstly restraining all the system but the ligand (*p*NP-Fuc), secondly restraining only the protein atoms (except hydrogen atoms) and a third step without restraints. Once the system was relaxed, an MD simulation was performed under periodic boundary conditions (PBC). The MD protocol consisted in a heating simulation (from 0 to 300 K) during 200 ps under NVT conditions using Langevin dynamics. Following, an equilibration period of 400 ps was performed. The first 200 ps were run under NPT conditions to reach a system density of around 1 g cm^−3^ and using a isotropic weak-coupling algorithm and the Berendsen barostat (Berendsen et al., 1984) at 1 atm. Then, the system volume was fixed and the next 200 ps were run under NVT conditions. An equilibration of 10 ns and the final production run of 100 ns were performed at 300 K using the NVT ensemble without any restraints. Along the MD, the covalent bonds containing hydrogen were constrained using the SHAKE algorithm (Ryckaert et al., 1977), and the particle-mesh Ewald method (Essmann et al., 1995) was used to treat long-range electrostatic interactions. A 1fs time step was used in all the MD trajectories. The analysis of the MD simulation was carried out using the standard tools of the AMBER 16 package (Case et al., 2005) [cpptraj program (Roe and Cheatham, 2013)] and VMD (Humphrey et al., 1996). Based on this analysis, we established the criteria for the selection of a few frames of the MD trajectory that served as starting point for the QM/MM calculations of the glycosylation (G) reaction.

To obtain the starting structure for the QM/MM calculations of the hydrolysis reaction (H), the product geometry of the glycosylation process was modified by deleting all the *p*NP coordinates except those of the just formed hydroxyl group that was converted into a water molecule using the PyMol program. The system was solvated with a cubic box of TIP3P water molecules, and their coordinates were relaxed at the MM level by constraining all protein atoms and the just built water molecule (acceptor). This was followed by a short MD simulation (210 ps) to allow the rearrangement of water molecules in the active site. From the last frame of that MD trajectory a QM/MM minimization was carried out to locate a reactant minimum to initiate the hydrolysis reaction.

For the transglycosylation reaction (T), the coordinates of the above mentioned CTT substrate were used as template to build those of the BnON(Me)-Glc acceptor substrate, enforcing the 4-OH hydroxyl group from the glucose moiety to overlay with the hydroxyl coordinates of the *p*NP leaving group from the previous glycosylation step. Three water molecules were deleted due to clashes with the acceptor molecule. The system was relaxed as done for the hydrolysis reactant.

All simulations were carried out using AMBER16 software (GPU (CUDA) version of the PMEMD (Götz et al., 2012; Salomon-Ferrer et al., 2013) package.

### QM/MM Calculations

**Figure 2:**
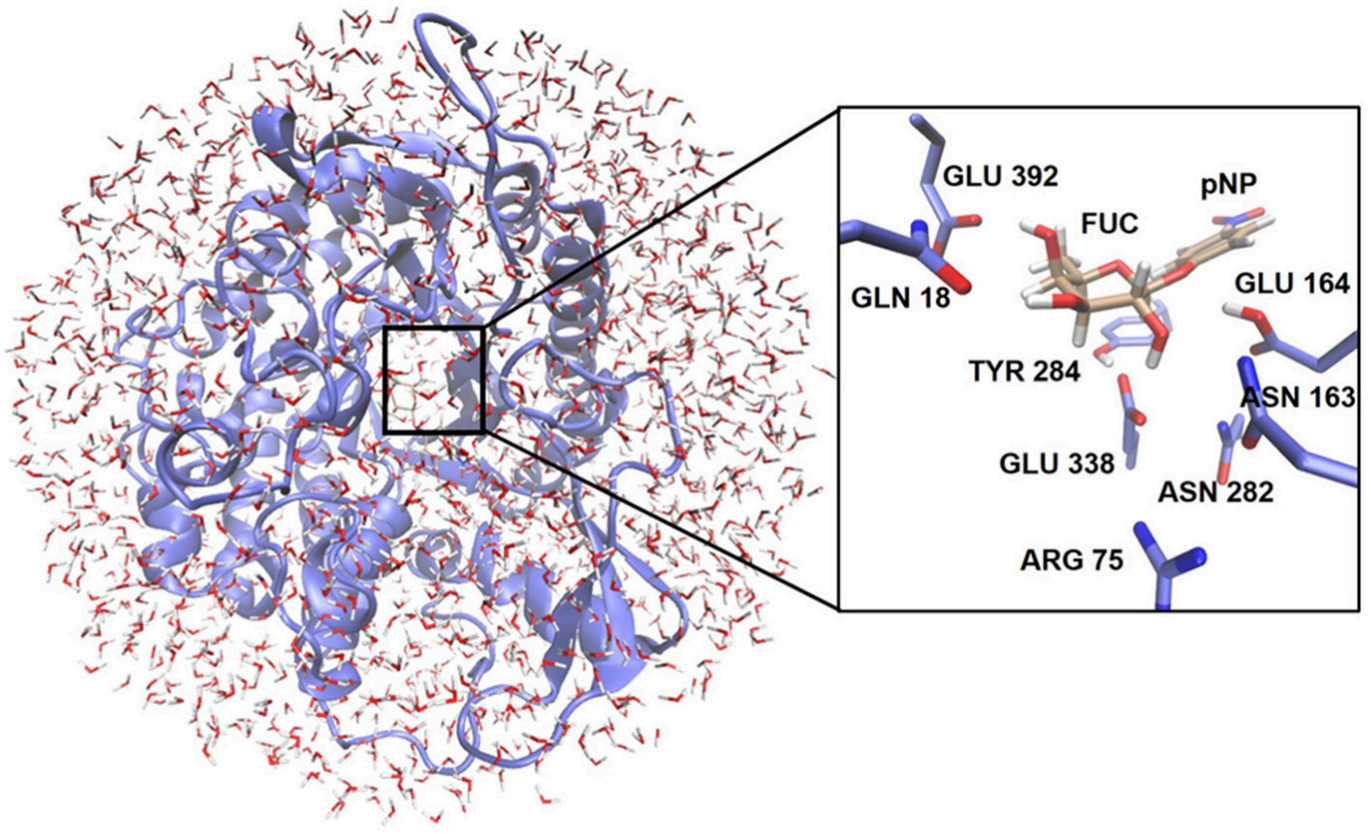
Representation of the enzymatic model used for the QM/MM calculations. The location of the QM region is highlighted. The inset shows as an example the arrangement of the QM atoms for the glycosylation reaction at the active site.

QM/MM calculations were performed with the modular program package ChemShell (Sherwood et al., 2003; Metz et al., 2014) using TURBOMOLE-V6.3 (Ahlrichs et al., 1989) to obtain the QM energies and gradients at the DFT level. The PBE0 functional (Adamo and Barone, 1999) was used, as it gave errors smaller than 0.5 kcal/mol in a recent benchmarking modeling glycosidic bond hydrolysis by glycosidases (Pereira et al., 2017) and inclusion of D3 correction did not affect significantly the calculated energies. MM energies and gradients were evaluated by DL_POLY (Smith and Forester, 1996), which was accessed through the ChemShell package, using the AMBER force field. The electrostatic embedding scheme (Bakowies and Thiel, 1996) was used within the QM/MM approach to let the MM point charges to polarize the electronic density of the QM region. No cutoffs were introduced for the nonbonding MM and QM/MM interactions. In all the QM/MM calculations, the cubic box of water molecules was simplified to a 30 Å sphere surrounding the full protein (Figure 2). All residues and water molecules within 15 Å from the anomeric center were included in the optimization process as active region (around 2,100 atoms) while the remaining atoms were kept fixed.

**Figure 3:**
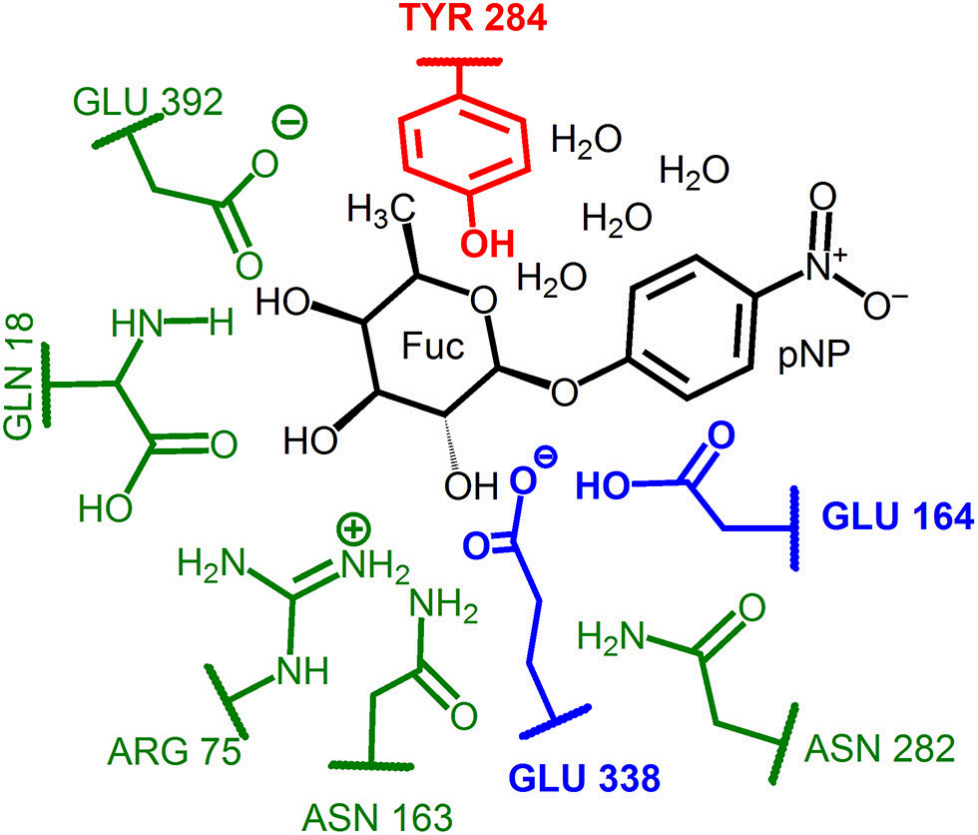
Representation of the QM regions used in the QM/MM study of the glycosylation reaction. The small QM region includes: the substrate atoms and water molecules (in black), the catalytic residues (in blue) and the Tyr184 residue (in red). For the hydrolysis and transglycosylation the O-*p*NP group was substituted by a water molecule and the BnON(Me)-Glc acceptor substrate, respectively. Three additional waters were included for the hydrolysis and just one for the transglycosylation step. The large QM region comprises for the three reaction steps the corresponding small QM region plus the protein residues depicted in green.

Two different QM regions were studied (Figure 3): the small QM region containing 84 atoms in the glycosylation step (the *p*NP-Fuc substrate, four nearby waters, and the side-chains of Glu164, Glu338, and Tyr284), and a large QM region with 138 atoms (including the small QM region and, in addition, the side-chains of Asn282, Asn163, Arg75, Gln18, and Glu392). For the hydrolysis and transglycosylation QM/MM descriptions the O-*p*NP group was substituted in the QM regions by a water molecule and the BnON(Me)-Glc molecule, respectively. Three water molecules were added (besides the nucleophilic water, WAT431) in the QM zones for the hydrolysis calculations whereas only one QM water molecule was left in the transglycosylation study. The number of QM atoms in the small/large regions for the hydrolysis and transglycosylation steps are 67/121 and 100/154, respectively. The methodology based on link atoms (three in the QM(small)/MM models and eight in the QM(large)/MM partitions) was used to define the QM/MM boundary with the charge-shift approach (Claeyssens et al., 2006). The total charge of both QM regions is−1 in the three catalytic reaction steps.

QM/MM optimizations were carried out employing the limited-memory Broyden-Fletcher-Goldfarb-Shanno (L-BFGS) (Liu and Nocedal, 1989) algorithm combined with the Hybrid Delocalized Internal Coordinate Scheme (Billeter et al., 2000) as implemented in Chemshell (Metz et al., 2014). Reaction paths were scanned by performing harmonically restrained optimizations along a properly defined reaction coordinate for each chemical process in steps of 0.2 Å. Reaction coordinates were defined as linear combinations of the three main distances involved in each step, a definition that we have successfully used when modeling other carbohydrate-active enzymes (Gómez et al., 2015). Thus, RC = [*d*(C1_FUC_-O4D_pNP_)-*d*(C1_FUC_-OE2_GLU338_)-*d*(H_GLU164_-O4D_pNP_)] for glycosylation, RC = [*d*(C1_FUC_-OE2_GLU338_)-*d*(C1_FUC_-O_WAT431_)-*d*(H1_WAT431_-OE2_GLU164_)] for hydrolysis and RC = [*d*(C1_FUC_-OE2_GLU338_)-*d*(C1_FUC_-O4_GLC_)-*d*(H4O_GLC_-OE2_GLU164_)] for transglycosylation. For each reaction step, the structure corresponding to the maximum of the potential energy profile was taken as indicative of the corresponding transition state. The PBE0 hybrid functional and two basis sets (SVP and TZVP) were used for the description of the small QM region, whereas the large QM region was always treated at the PBE0/TZVP level. In addition, QM(PBE0/TZVP)/MM single point energy calculations on the QM(PBE0/SVP)/MM geometries along the hydrolysis and transglycosylation potential energy profiles were also performed.

Natural population analysis (NPA) charges (Reed et al., 1985) were determined from QM/MM calculations with the QM region described at the PBE0/TZVP level. The contribution of the different residues to the QM/MM potential energy barriers of the hydrolysis and transglycosylation steps was examined by setting their point charges to zero in additional single point energy calculations at the QM(PBE0/TZVP)/MM level along the QM(PBE0/TZVP)/MM reaction paths.

Umbrella sampling at the QM(SCC-DFTB)/AMBER level was performed to compute the free energy profile for the hydrolysis and transglycosylation steps, using the dynamics module within ChemShell. The reaction coordinates, defined as before, were scanned at 0.1 Å intervals using a harmonic force constant of 300 kcal/mol·Å^2^. The same force constant was used for restraining the distance between OE1_GLU338_ and O2_FUC_ to 2.4 Å in the hydrolysis step to correct for undesired proton transfers occurring during the simulations. Still, some of the hydrolysis data had to be discarded in the analyses presented below. Forty-five windows were used for hydrolysis and fifty for transglycosylation. Each simulation consisted of 20 ps of equilibration and 60 ps of production/data collection, under the NVT ensemble and using the Nosé-Hoover thermostat (Nosé, 1984; Hoover, 1985). All atoms from residues beyond 20 Å of C1_FUC_ were frozen. The histograms show sufficient overlap of the windows for the chosen stepsize and force constant. The umbrella integration analysis method (Kästner and Thiel, 2005, 2006) was used to compute the free energy profiles.

## Results and Discussion

### Glycosylation Step

#### Molecular Dynamics Simulations

To obtain a first structural insight into the dynamics of the Ttβ-gly/*p*NP-Fuc Michaelis complex, the root-mean-square deviations (RMSDs) of the protein backbone and of the heavy atoms of the *p*NP-Fuc substrate were calculated along the 100 ns of production trajectory (Supplementary Figure S1). The protein structure is well equilibrated. The substrate RMSD (with an average value of 0.78 ± 0.37 Å) presents an oscillatory pattern because the *p*NP phenyl group is flipping between two conformations all along the trajectory. As it will be detailed below, the Fuc ring is more rigidly bound at the−1 subsite than the *p*NP group is at the larger +1 subsite, thus having this latter moiety of the substrate more room to move along the simulation.

To analyze in more detail the molecular interactions more likely to play a role in the catalytic mechanism, a search of the most populated hydrogen bonds between the substrate, protein residues and water molecules at the active site was carried out along the MD simulation. In Table 1 a summary of these hydrogen bonds is presented (a more complete analysis is given in Supplementary Tables S2–S5). The fraction of frames the hydrogen bond is present (the H-bond occupancy), along with the corresponding donor···acceptor average distance, are given. An occupancy higher than 90% is obtained for the interaction between the carboxylate oxygen OE1 of the catalytic acid/base residue Glu164 and HD22 of Asn282. This interaction helps to maintain (with an occupancy of 90.6%) the hydrogen bond between the proton of Glu164 and the glycosidic oxygen (O4D_pNP_). This is a very important interaction for the glycosylation process because in this way Glu164 remains well-positioned (average heavy atoms distance of 2.76 Å) for proton donation to the leaving group. The catalytic nucleophilic residue Glu338 mainly interacts through its carboxylate oxygen OE2 with Tyr284 (88.9% occupancy) so that the negative charge on the nucleophile becomes stabilized. In addition, this same carboxylate group of the nucleophilic residue Glu338 (through its OE1 oxygen) establishes nearly half of the time another hydrogen bond with the glycosyl 2-OH group of the substrate. Some oscillation of this 2-OH group between OE1_GLU338_ (46.6% occupancy) and OE2_Glu338_ (16.3% occupancy) is actually observed. As commented in the Introduction, this interaction is key for the stabilization of the glycosylation transition state, especially for retaining β-glycosidases because the interaction with the nucleophile will affect oxocarbenium cation formation. Moreover, OE1_Glu338_ is also H-bonded with Arg75 side chain and a water molecule. On the other hand, the 2-OH group of the Fuc ring also interacts with Asn163 side-chain (with an occupancy of 46.8%). The 3-OH group of Fuc establishes a H-bond with HE2_HIS119_ (34.4% occupancy) and with OE1_GLN18_ (79.3%). The 4-OH and 5-OH of Fuc are hydrogen bonded as acceptors to the same water molecule (with an occupancy of around 20% for each Fuc···water interaction), and the axial 4-OH of Fuc is H-bonded to OE1_GLU392_ (62.5% occupancy). This latter interaction structurally differs from that reported by Badieyan et al. (2012) when cellobiose is hydrolyzed by Osβ-gly, as in Glc this hydroxyl group is in the equatorial position, which makes it interact with the other oxygen of the Glu392 carboxylate.

**Table 1:** Analysis over the MD simulation of the most populated hydrogen-bonds between *p*NP-Fuc, protein residues, and water molecules at the active site.

**Acceptor**	**Donor**	**H-bond** **occupancy %**	**Average** **Distance (Å)**
O_NO2pNP_	Solvent	99.6	2.79
OE1_GLU164_	ND2_ASN282_	92.5	2.81
O4D_pNP_	OE2_GLU164_	90.6	2.76
OE2_GLU338_	OH_TYR284_	89.0	2.76
OE1_GLN18_	O3_FUC_	79.3	2.72
OE1_GLU392_	O4_FUC_	62.5	2.80
O2_FUC_	ND2_ASN163_	46.8	2.90
OE1_GLU338_	O2_FUC_	46.6	2.84
O3_FUC_	NE2_HIE119_	34.4	2.87
O5_FUC_	Solvent	24.0	2.85
O4_FUC_	Solvent	20.4	2.84
O3_FUC_	NE1_TRP393_	16.6	2.91
OE2_GLU338_	O2_FUC_	16.3	2.85
OE1_GLU338_	NH1_ARG75_	13.6	2.91

*H-bond occupancies are defined as the fraction of frames the bond is present. Only H-bond occupancies above 10% are included. The average donor to acceptor heavy atoms distances of the bonds when present are also given*.

To summarize, the glycosyl moiety of the substrate bound at the−1 subsite establishes a complex H-bond network with the protein and some water molecules that contributes to its stabilization. It is worth highlighting that all the first- and second-shell protein residues around the−1 subsite identified in this H-bond analysis are highly conserved residues among the GH1 family (Bráa et al., 2010; Camargo et al., 2019). In contrast, the H-bond analysis did not identify any conserved residue around the +1 site so confirming the lack of specificity for the aglycone moiety within the GH1 family as previously observed (Bráa et al., 2010). The *p*NP molecule only establishes H-bonds with two water molecules all along the MD trajectory through its NO2 group. In addition to those H-bond interactions, the Fuc ring remains surrounded all along the trajectory by hydrophobic interactions established with conserved residues such as Trp385, Trp393, Trp120, and also Phe401, whereas the *p*NP leaving group basically interacts with Trp312.

To initiate the QM/MM study of the glycosylation catalytic mechanism, two MD snapshots of the Ttβ-gly/*p*NP-Fuc Michaelis complex were selected. Those structures were filtered by imposing the presence of the direct H-bonds (O4D_pNP_-H_GLU164_ and OE2_Glu338_-H2O_FUC_) between the two catalytic residues, Glu338 and Glu164, with the *p*NP-Fuc substrate. In addition, the presence of the H-bond between Tyr284 and Glu338 was also verified in the selected frames. The relevance of this interaction (and of describing it at the QM level) for the energetics of the glycosylation step and hydrolysis steps has been recently highlighted by Badieyan et al. (2012) in their QM/MM study of Osβ-gly mentioned above. This is the reason why we have included Tyr284 in the QM(small)/MM partition. Finally, in the two frames selected it was also verified that the additional predominant H-bond interactions highlighted in Table 1 were also present.

#### QM/MM Calculations

In this section the results of the QM/MM calculations on the glycosylation reaction will be presented. This catalytic step consists in the cleavage of the glycosidic bond of the *p*NP-Fuc molecule to form a covalent glycosyl-enzyme intermediate with Glu338. Despite *p*NP is a good leaving group, acid catalysis is required for this mechanistic step, a role that is carried out by the protonated Glu164 (Figure 1).

The two selected frames from the MD trajectory (denoted as G_I_ and G_II_ in this paper) were optimized using the two QM/MM partitions described above and the corresponding minima were located and characterized. From those minimum energy structures, the potential energy profiles along the glycosylation step were calculated as a function of the reaction coordinate RC = [*d*(C1_FUC_-O4D_pNP_)-*d*(C1_FUC_-OE2_GLU338_)-*d*(H_GLU164_-O4D_pNP_)]. In Table 2 the QM/MM potential energy barriers (obtained from the corresponding energy profiles) and the reaction energies (considering the optimized glycosyl-enzyme product) for the glycosylation step at the QM = PBE0/SVP and PBE0/TZVP levels using the QM(small)/MM and QM(large)/MM partitions are given for both frames. The corresponding energy values obtained by means of PBE0/TZVP single-point energy calculations on the PBE0/SVP(small)/MM geometries are also given. It can be observed that the potential energy barrier using the QM(small)/MM partition takes values in between 28 and 30 kcal/mol when the smaller basis set is used. The energy correction using the larger basis set tends to increase those energy barriers (by 0.4–2 kcal/mol) especially for the G_II_ frame. However, when the geometry optimization is carried out with the TZVP basis set, no significant differences are obtained with respect to the SVP energy values. These energy barriers are in the range of that calculated with a large QM region for the glycosylation reaction involving cellobiose and Osβ-gly (Badieyan et al., 2012), and also of those calculated for other retaining GHs (Petersen et al., 2009, 2010; Biarnés et al., 2011). However, a large effect is observed in the present system when the large QM region is used with the TZVP basis set. Lower potential energy barriers (by 5.9 and 9.8 kcal/mol for frames G_I_ and G_II_, respectively) were obtained for both frames. Notice that, as *p*NP is a better leaving group than the Glc of cellobiose modeled in the glycosylation reaction catalyzed by Osβ-gly (Badieyan et al., 2012), a lower energy barrier should be expected. In particular, the potential energy barriers calculated at QM(large)/MM level and the TZVP basis set are 22.0 kcal/mol (for G_I_) and 20.4 kcal/mol (G_II_). These values are in better and in reasonable qualitative agreement with the value of 17.1 kcal/mol for the phenomenological free energy of activation derived from the experimental *k*_*cat*_ at 40°C (Teze, 2012). The significant differences between the calculated potential energy barriers reflect that they are very sensitive to different QM/MM partitioning schemes and that non-catalytic residues (other than Tyr284) might play a role in the stabilization of the glycosylation transition state, as previously stated. As for the reaction energies, the glycosylation process turns out to be more endoergic for frame G_I_ than for frame G_II_ when the QM(small)/MM partition is used. However, both glycosylation reactions become clearly endoergic when the QM region is enlarged. Interestingly, the endothermic nature of the glycosylation reaction was recently observed in previous studies where their authors suggest that an endothermic glycosylation might be a prerequisite for efficient transglycosylation (Raich et al., 2016).

**Table 2:** QM/MM potential energy barriers (ΔV^≠^) and reaction energies (ΔV_R_) calculated for the glycosylation step for the two complexes studied (G_I_ and G_II_).

	**G**_****I****_		**G**_****II****_	
**QM level**	**ΔV^≠^**	**ΔV_R_**	**ΔV^≠^**	**ΔV_R_**
PBE0/SVP^a^	28.4	7.1	30.2	0.4
PBE0/TZVP//PBE0/SVP^a^	28.8	5.1	32.1	−0.1
PBE0/TZVP^a^	27.9	6.3	30.2	0.6
PBE0/TZVP^b^	22.0	4.8	20.4	6.6

a *QM(small)/MM*.

b *QM(large)/MM*.

*Energies are given in kcal/mol*.

**Figure 4:**
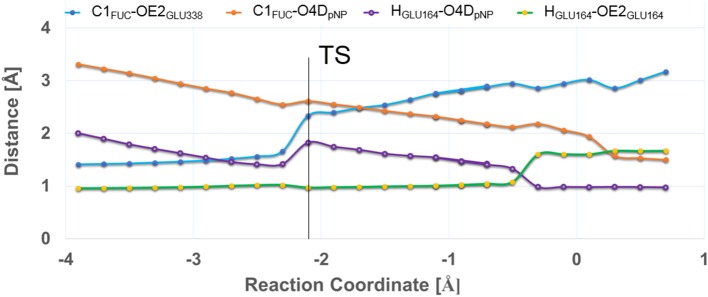
Evolution of C1_FUC_-O4D_pNP_ (in blue), C1_FUC_-OE2_GLU338_ (in orange), H_GLU164_-O4D_pNP_ (in purple), and H_GLU164_-OE2_GLU164_ (in green) distances (in Å) along the glycosylation reaction. The results correspond to frame G_I_ calculated with the QM(large)/MM partition and at the QM(PBE0/TZVP) level for the QM region.

**Figure 5:**
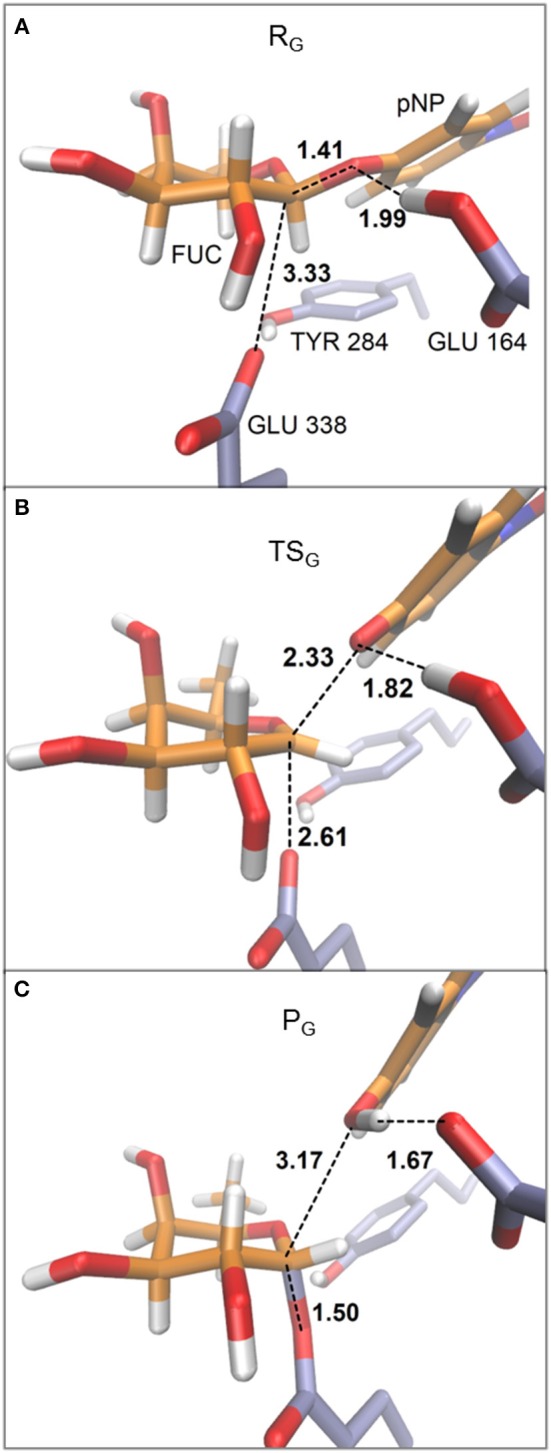
Representative structures for reactant **(A)**, transition state **(B)**, and product **(C)** for the glycosylation reaction. The depicted geometries correspond to frame G_I_ within the QM(large)/MM partition and with the QM region described at the PBE0/TZVP level. Distances are in Å.

In Figure 4 the evolution of the four main interatomic distances involved in the glycosylation reaction (C1_*FUC*_ - O4D_pNP_, C1_FUC_ - OE2_GLU338_, H_GLU164_ - O4D_pNP_, and H_GLU164_ - OE2_GLU164_) are plotted for the G_I_ frame along the reaction coordinate calculated with the QM(large)/MM partition and at the PBE0/TZVP level for the QM region. In Figure 5 the molecular representation of the corresponding reactant, transition state and product are depicted. It can be observed that the C1_FUC_ - OE2_GLU338_ distance between the nucleophile residue and the anomeric carbon gradually diminishes from 3.33 Å at reactants (RC = −3.90 Å) to a value of 2.61 Å at the transition state (RC = −2.10 Å) (what corresponds to a bond not yet formed), and finally takes a value of 1.50 Å at the covalent glycosyl-enzyme intermediate (RC = 0.70 Å) in which this bond is already completely formed. The length of the glycosidic bond C1_FUC_ - O4D_pNP_ is 1.41 Å at the reactants, increases to 2.33 Å at the TS (what corresponds to the glycosylic bond already broken) and finally takes a value of 3.17 Å at the product structure in which the leaving group has been released. For the second frame (G_II_) the distances are very similar (Supplementary Table S6). These structural results are coherent with the dissociative nature of the glycosylation transition state. The oxocarbenium character of this TS is also made apparent by a higher degree of double bond character of the C1_FUC_-O5_FUC_ bond (the C1_FUC_-O5_FUC_ distance diminishes 0.14 Å from the reactant to the TS structure for both frames). In addition, the acidic residue Glu164 gradually approaches the glycosidic bond along the two reaction paths (the O4D_pNP_ - H_GLU164_ hydrogen bond diminishes from 1.99 Å (G_I_) and 2.02 Å (G_II_) at the reactants to 1.82 and 1.88 Å at the G_I_ and G_II_ TS, respectively) although the proton is not yet transferred. Proton transfer takes place when the OE2_GLU338_-C1_FUC_ distance is ~2 Å, and is followed by complete OE2_GLU338_-C1_FUC_ bond formation. This is consistent with a change in pK_a_ of Glu164 that makes proton transfer easier as the negative charge of the leaving group gradually increases and that of the nucleophile gradually decreases with the progress of the glycosylation reaction. When a smaller QM region is used within the QM/MM approach, and independently of the basis set employed for the QM description, the transition state can also be described as dissociative (Supplementary Table S7 and Supplementary Figure S2). Curiously, the O4D_pNP_ - H_GLU164_ H-bond distance at the reactant varies by 0.42 Å between the two frames considered (G_I_ and G_II_), and a significant difference in this distance is also observed at the corresponding TSs. When applying the QM(large)/MM partition, these differences between frames disappear. With the smaller QM region, the nucleophilic attack by Glu338 is completed at lower RC values than with the QM(large)/MM partition and quite before the proton is fully transferred. Thus, the description at the QM level of the H-bonds that Glu338 and Glu164 establish with their neighboring residues seems to affect significantly their nucleophilic and acid/base characteristics.

The inclusion of other protein residues besides the catalytic ones (Glu338, and Glu164) and Tyr184 in the zone treated quantum-mechanically (as in our QM(large)/MM model) also affects the stabilization by the enzyme environment of the oxocarbenium ion and the negatively charged glycosyl oxygen anion. Moreover, as mentioned in the Introduction, one of the most stabilizing protein-substrate interactions at the glycosylation TS is the H-bond between the 2-OH group of the sugar moiety and the nucleophilic residue Glu338. This stabilizing contribution correlates with a reduction of the OE1_GLU338_ - H2O_FUC_ H-bond distance, from 2.09 Å and 2.37 Å at reactants to 1.82 and 1.85 Å at the TS of G_I_ and G_II_, respectively (Supplementary Table S6). The Fuc at the −1 subsite is also stabilized along the glycosylation pathway by the HD21_ASN163_ - O2_FUC_ interaction as the corresponding H-bond distance diminishes around 0.10 Å from reactants to the transition state structures. Tyr284 plays a relevant role along the glycosylation process. As the OE2_GLU338_ atom loses negative charge upon formation of the C1_FUC_ - OE2_GLU338_ bond, the Tyr284 hydroxyl group separates from Glu338 and clearly approaches (with a reduction in the bond distance of around 1.3 Å at the TSs and of around 1.9 Å at the product structures) the O5_FUC_ atom. The expected oxocarbenium ion-like character of the TS is favored by the coplanarity of the C2, C1, O5, and C5 atoms of the fucose ring which adopts a slightly distorted chair conformation close to a half-chair (^4^H_3_) and with some ^4^E character.

At this point it is interesting to comment that it has been shown that GHs usually bind the substrate in a subtle but critical distorted conformation (Davies et al., 2012). Such conformational change brings the glycosidic bond closer to the equatorial orientation and to a more planar ring structure (so closer to the oxocarbenium TS geometry) that facilitates in-line nucleophilic attack. A possible exception to this has been recently reported for family GH3 enzymes, for which the glucose bound at the−1 subsite seems to adopt a ^4^C_1_ conformation (maybe slightly distorted to ^4^H_5_) in the Michaelis complex according to QM/MM calculations and crystallographic data (Hrmova et al., 2001; Geronimo et al., 2018). Different conformational itineraries (from reactants to TS and to products) have been associated with different GHs (Ardèvol and Rovira, 2015). Thus, for retaining β-glucosidases acting on glucose substrates the ^1^S_3_ → ^4^H_3_ → ^4^C_1_ itinerary has been described for the glycosylation step. The QM/MM study by Badieyan et al. (2012) of the glycosylation step catalyzed by rice Osβ-gly using cellobiose as substrate agrees with this itinerary. However, in the present study, in which we have *p*NP-Fuc instead of Glc(β1-4)β-Glc as substrate, we were unable to characterize a Michaelis complex in a ^1^S_3_ conformation. The QM(large)/MM optimized reactant has a slightly distorted ^4^C_1_ conformation (with θ and ϕ angles values of 216 and 27°, respectively). Attempts to obtain a different conformation by maintaining the ring restrained previous to the full QM(large)/MM minimization only produced an alternative minimum close to a ^4^E (slightly ^4^H_3_) conformation. This structure presents a small increase in the C1_FUC_ – O4D_pNP_ distance (1.49 Å) and slightly shorter C1_FUC_ – OE2_GLU338_ (3.29 Å), C1_FUC_ – O5_FUC_ (1.36 Å), and H_GLU164_ – O4D_pNP_ (1.77 Å) distances than the previous one. The potential energy profile calculated from this minimum gives a barrier height of only 10 kcal/mol (Supplementary Figure S3), which is much smaller than the experimental value. This suggests that, although such conformation may be possible, it may not be the most favorable conformation that the enzyme will bind and, thus, it has no catalytic significance. Badieyan et al. (2012) reported the importance of having Glu440 of Osβ-gly (that interacts with the 4-OH and 6-OH groups of Glc) in the QM region in order to characterize the ^1^S_3_ conformation. In the present study, the QM(large)/MM partition includes the equivalent Glu392 residue, amongst many others. At the origin of these differences may be the fact that we are modeling Fuc instead of Glc (they differ in the position of the 4-OH group and Fuc has no 6-OH group but a methyl in this position); or that our substrate is not an oligosaccharide but a synthetic phenyl derivative that may introduce less steric requirements than a monosaccharide at the +1 subsite. Nevertheless, as glycosidic bond breakage occurs early in the reaction, the ring is distorted early along the calculated reaction coordinate with subsequent attack by the nucleophilic Glu338 occurring in the expected orientation.

### Hydrolysis and Transglycosylation Steps (Deglycosylation)

Once the *p*NP molecule leaves the enzyme active site, a water molecule can compete as nucleophile in a hydrolysis reaction with the acceptor ligand (BnON(Me)-Glc), that also attacks as a nucleophile on the anomeric carbon of the fucose molecule in a transglycosylation reaction leading to the *N*-methyl-*O*-benzyl-*N*-(β-*D*-fucopyranosyl(1-4)β-*D*-glucopyranosyl)-hydroxylamine product. The attacking water molecule as well as the BnON(Me)-Glc ligand are activated by a proton transfer to the Glu164 residue that acts as a base along those two nucleophilic processes.

Starting from the minimized MD structure (see Methodology section) in which a water molecule (WAT431) was located at the active site, close enough to the anomeric carbon of fucose ring, we calculated the QM/MM energy profile for the hydrolysis process using a reaction coordinate defined as RC = [*d*(C1_FUC_-OE2_GLU338_)-*d*(C1_FUC_-O_WAT431_)-*d*(H1_WAT431_-OE2_GLU164_)]. As mentioned above, a H-bond network of three water molecules, in addition to the nucleophilic WAT431, were included in the QM region for the hydrolysis simulation. On the other hand, for the transglycosylation step, initial energy profiles starting from minimized MD structures gave highly overestimated energy barriers (>34 kcal/mol), suggesting some structural deficiencies in the model. With the aim of improving this model, we followed a strategy successfully used by our group in previous studies of enzyme catalyzed reactions involving sugar-transfer between donor and acceptor substrates (Albesa-Jové et al., 2015; Mendoza et al., 2016, 2017). The refinement protocol consists on running a QM(SCC-DFTB)/MM MD of, in this case, the transglycosylation product, followed by scan calculations of frames from this MD to generate new models for the transglycosylation reactant that is then minimized at the QM(PBE0)/MM level. By doing this, interactions between donor and acceptor (or also involving water molecules) that are difficult to capture in the MM MD seem to be better represented. In the present system, the structural changes observed upon refinement involve the reorientation of the 4-OH hydroxyl of the acceptor Glc toward the Fuc ring. This displaces a water molecule that was located between the two and that most likely hindered acceptor approach during reaction (Supplementary Figure S4). In the new reactant structure, the Glc hydroxyl will be able to better interact with Fuc as transglycosylation takes place. Substrate-substrate interactions have been shown to have a very important contribution in the sugar transfer catalyzed by retaining glycosyltransferases (Gómez et al., 2012, 2013; Mendoza et al., 2017) and could, thus, be also relevant for transglycosylation. Two frames (called here T_I_ and T_II_) were generated by this approach. From the two optimized QM/MM reactant structures, the transglycosylation potential energy profiles for the corresponding β(1-4) glycosidic linkages were calculated using as reaction coordinate the following expression RC = [*d*(C1_FUC_-OE2_GLU338_)-*d*(C1_FUC_-O4_GLC_)-*d*(H4O_GLC_-OE2_GLU164_)]. In this model only one water molecule remains in the QM region. We did not analyze other glycosidic linkages between the Fuc and BnON(Me)-Glc because it has been proven experimentally that the native enzyme does not produce other product regioisomers when these two substrates are used (Teze et al., 2013).

In Table 3 the potential energy barriers and the reaction energies corresponding to the hydrolysis and the transglycosylation steps are given for the two QM/MM partitions used in this study and at the different QM levels of electronic structure theory analyzed. The first general observation is that the potential energy barrier for the hydrolysis step is very similar to that of the transglycosylation one, as expected because the experimental result for this reaction catalyzed by WT Ttβ-gly gives a transglycosylation yield of 36%, which implies differences in the energy barriers no larger than 1 kcal/mol. With the small QM region at the PBE0/SVP level, the hydrolysis potential energy barrier is 2.1 and 0.9 kcal/mol higher than for the transglycosylation frames T_I_ and T_II_, respectively. According to this result, the transglycosylation reaction would be faster than the hydrolysis process. This trend is inverted when single-point energy calculations are carried out at a higher level (QM(PBE0/TZVP)), being the hydrolysis energy barrier now slightly lower (by 0.7 (T_I_) and 0.9 (T_II_) kcal/mol). The correct trend between the hydrolysis and transglycosylation barriers is confirmed when a bigger basis set is used in the QM/MM optimizations and also when the QM region is enlarged. As for the glycosylation step, the values of the energy barriers calculated with the smaller QM region are around 6 kcal/mol higher than those obtained with the larger QM region. Those energy differences reflect again that the two reaction processes are very sensitive to different QM/MM partitioning schemes. Despite this, the difference between transglycosylation and hydrolysis energy barriers is similar (1.2 kcal/mol for T_I_ and 2.6 kcal/mol for T_II_). Thus, the energy trends might be reproduced at a lower computational cost (QM(PBE0/TZVP)/MM but QM(small)/MM). As for the reaction energies, the hydrolysis process is more exoergic than the transglycosylation reaction. This result is in agreement with previous results by Bráa et al. (2010) in their study of the catalytic mechanism of a β-galactosidase and with the known thermodynamically favored hydrolysis over synthesis reaction (Planas and Faijes, 2002).

**Table 3:** Potential energy barriers (ΔV^≠^) for the hydrolysis (H) and transglycosylation processes (T_I_ and T_II_, corresponding to the two different frames studied) along with the corresponding reaction energies (ΔV_R_) with the two QM/MM partitions and at the different QM levels employed.

	**PBE0/SVP^a^**		**PBE0/TZVP//PBE0/SVP^a^**		**PBE0/TZVP^a^**		**PBE0/TZVP^b^**	
	**ΔV^≠^**	**ΔV_R_**	**ΔV^≠^**	**ΔV_R_**	**ΔV^≠^**	**ΔV_R_**	**ΔV^≠^**	**ΔV_R_**
H	25.4	−16.7	22.7	−17.8	24.6	−15.8	18.3	−17.4
T_I_	23.3	−9.7	23.4	−8.5	25.6	−8.3	19.5	−12.5
T_II_	24.5	−8.9	23.6	−4.6	27.9	−5.8	20.9	−4.0

a *QM(small)/MM*.

b *QM(large)/MM*.

*All energies are given in kcal/mol*.

**Figure 6:**
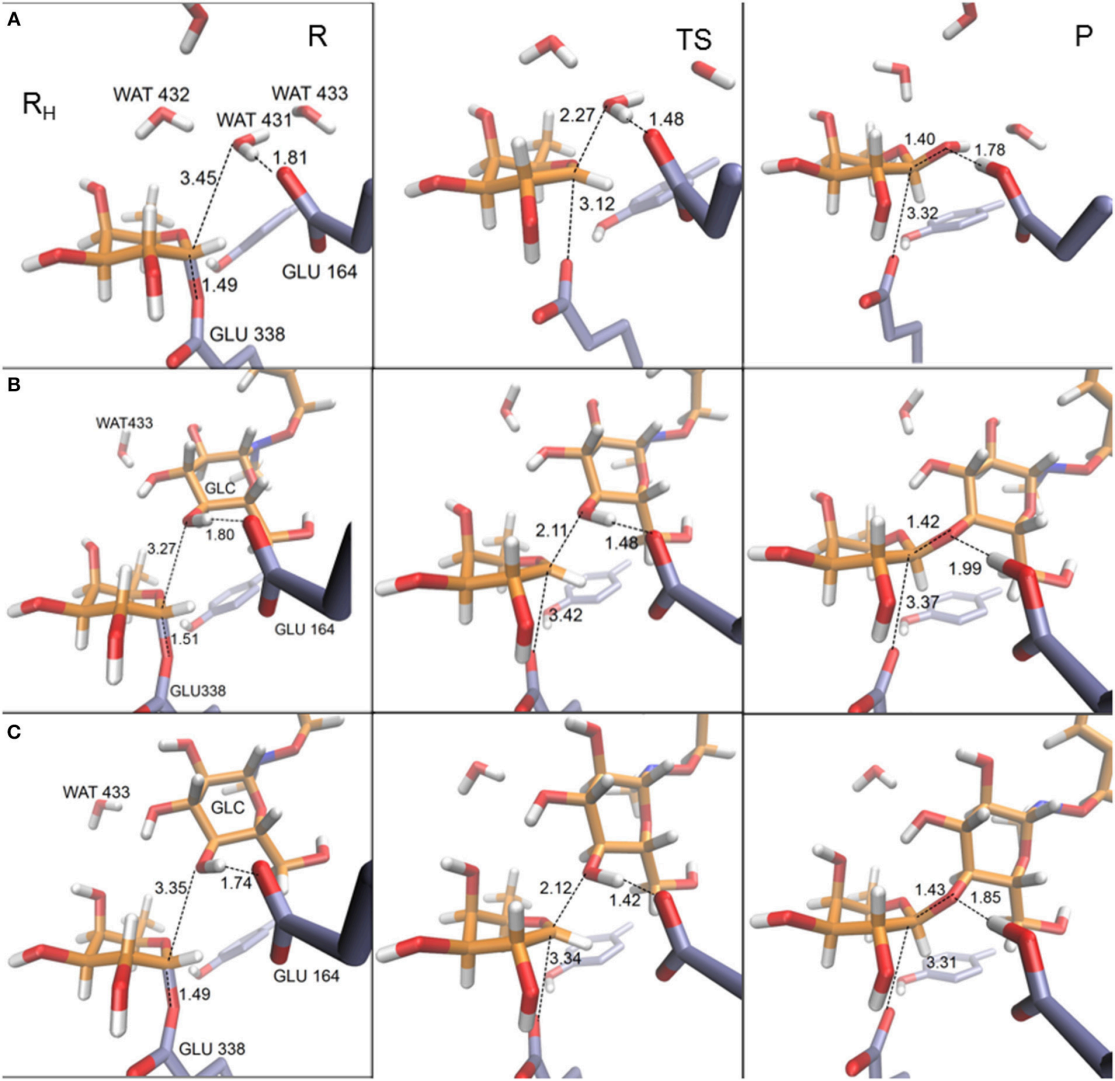
Representative structures for reactant (R), transition state (TS), and product (P) for hydrolysis **(A)** and transglycosylation reactions (frame T_I_ **(B)** and frame T_II_ **(C)**. The depicted geometries were calculated within the QM(large)/MM partition and with the QM region described at the PBE0/TZVP level. Distances are in Å.

In Table 4 the most relevant interatomic distances (calculated with the QM(large)/MM at the QM(PBE0/TZVP)/MM level) along the hydrolysis and transglycosylation pathways are presented and the corresponding molecular structures of the different reactants, transition states and products are depicted in Figure 6 (for QM(small)/MM results see Supplementary Table S8). At reactants, with QM(large)/MM, the initial location of the water molecule in the hydrolysis process corresponds to a slightly more distant position from the anomeric carbon (3.45 Å) than in the two reactants of the transglycosylation process (3.27 and 3.35 Å). The hydrolysis values reported for Glc hydrolysis by rice β-glucosidase (Osβ-gly) are slightly larger (3.62–3.75 Å) (Badieyan et al., 2012; Wang et al., 2013), as occurs at the same level of theory when the QM(small)/MM partition is used (Supplementary Table S8). Also at TS structures the C1_FUC_-O_Acc_ bond, that is still forming, is slightly longer for hydrolysis (with distances of 2.27, 2.11, and 2.12 Å at H, T_I_ and T_II_ transition states, respectively), while the nucleophile Glu338 side chain is completely displaced from the sugar in both reactions but slightly closer from C1_FUC_ in the case of hydrolysis (with C1_FUC_-OE2_GLU338_ distances of 3.12, 3.42, and 3.34 Å at H, T_I_, and T_II_ transition states, respectively). For Glc hydrolysis by Osβ-gly, slightly shorter distances have been reported, especially for the C1_GLC_-O_Nu_ one (1.89–2.14 and 2.45–2.51 Å, for C1_GLC_-O_Acc_ and C1_GLC_-O_Nu_, respectively) (Badieyan et al., 2012; Wang et al., 2013). Along the nucleophilic attacks, the proton from the water molecule or the BnON(Me)-Glc acceptor comes closer to the general base residue Glu164 but it has not been transferred yet at the transition states (H_Acc_-OE2_GLU164_ distances between 1.42 and 1.48 Å). This is in agreement with the hydrolysis description given on previous works (Badieyan et al., 2012; Wang et al., 2013; Geronimo et al., 2018). Interestingly, this proton transfer is more advanced in the hydrolysis and transglycosylation TSs that it was the Glu164 to *p*NP one in the glycosylation TS (*d*(H_GLU164_-O4D_pNP_ ~1.8Å). Notice that this is the first time that the transglycosylation step is studied for wild-type family GH1, thus direct comparison for this step with previous works is not possible. For family GH2, potential energy scans followed by TS characterization identified a dissociative TS for transglycosylation and with the proton not yet transferred; however, detailed structural information (i.e., distances) was not provided (Bráa et al., 2010). Besides, for the GH2 enzyme a magnesium ion interacting with the acid/base residue participates in catalysis, which is likely to introduce some structural differences when compared to the present system (e.g., the hydrolysis TS presented the anomeric C1 atom equidistant from the nucleophile and the attacking water (2.25 Å), which differs from all the results commented above).

**Table 4:** Distances (in Å) between selected atoms involved in each reaction step for the reactant, transition state (TS) and product of the hydrolysis (H) and transglycosylation (T_I_ and T_II_, corresponding to the two different frames studied) steps.

	**Reactant**			**TS**			**Product**		
	**H**	**T_**I**_**	**T_**II**_**	**H**	**T_**I**_**	**T_**II**_**	**H**	**T_**I**_**	**T_**II**_**
*d*(C1_FUC_-OE2_GLU338_)	1.49	1.51	1.49	3.12	3.42	3.34	3.32	3.37	3.31
*d*(C1_FUC_-O_Acc_)	3.45	3.27	3.35	2.27	2.11	2.12	1.40	1.42	1.43
*d*(C1_FUC_-O5_FUC_)	1.36	1.35	1.36	1.24	1.25	1.25	1.39	1.38	1.38
*d*(H_Acc_-OE2_GLU164_)	1.81	1.80	1.74	1.48	1.48	1.42	0.96	0.96	0.96
*d*(H_TYR284_-O5_FUC_)	2.08	2.19	2.13	3.00	3.22	3.19	3.94	3.98	3.81
*d*(H_TYR284_-OE2_GLU338_)	2.46	2.46	2.48	1.90	1.86	1.87	1.82	1.83	1.82
*d*/H_ARG75_-OE1_GLU338_)	2.19	2.25	2.25	2.09	2.27	2.10	2.05	2.11	2.08
*d*(H2O_FUC_-OE1_GLU338_)	1.78	1.78	1.75	1.89	1.84	1.75	2.05	2.15	1.92
*d*(H4O_FUC_-O_GLU392_)	2.29	2.73	4.13	2.16	2.56	4.10	1.93	2.00	4.00
*d*(H_ASN163_-O2_FUC_)	1.97	1.94	1.93	1.96	1.94	1.93	1.96	2.06	1.98
*d*(H3O_GLC_-O4_FUC_)	–	2.86	2.83	–	2.74	2.45	–	3.11	2.35
*d*(H3O_GLC_-O5_FUC_)	–	3.96	4.06	–	3.43	3.55	–	1.88	2.35
*d*(H3O_GLC_-O4_GLC_)	–	2.34	2.33	–	2.41	2.41	–	2.53	2.57
*d*(O3_GLC_-H2_WAT433_)	–	2.00	2.23	–	2.03	2.25	–	2.01	2.25
*d*(O_WAT431_-H2_WAT432_)	1.82	–	–	1.86	–	–	2.77	–	–
*d*(H2_WAT431_-O_WAT433_)	2.02	–	–	2.19	–	–	2.33	–	–
*d*(H2_WAT432_-O5_FUC_)	3.58	–	–	3.33	–	–	1.95	–	–
*d*(H1_WAT432_-O4 _FUC_)	2.28	–	–	2.25	–	–	2.59	–	–

*The results correspond to QM(large)/MM and at the PBE0/TZVP level. The Acc subscript refers to the acceptor water and glucose moieties in hydrolysis and transglycosylation processes, respectively*.

Despite the sensitivity observed for these TS geometries to the DFT functional, the basis set, the QM/MM partition, the characterization method, the substrates or the specific enzyme, it is clear that both the hydrolysis and the transglycosylation transition states are very loose structurally, and they have a dissociative nature. From the distances reported in Table 4, which are thus to be taken as orientative, some trends can be extracted at the QM(PBE0/TZVP)/MM with QM(large)/MM level. For example, the sum of the C1_FUC_-O_Acc_ and C1_FUC_-OE2_GLU338_ distances for the deglycosylation TSs (5.39, 5.53, and 5.46 Å for H, T_I_, and T_II_ transition states, respectively) are all significantly larger than the sum of the analogous C1_FUC_-O4D_pNP_ and C1_FUC_-OE2_GLU338_ distances at the glycosylation TS (which is 4.97 Å on average). On the other hand, the differences between C1_FUC_-O_Acc_ and C1_FUC_-OE2_GLU338_ distances in the deglycosylation TSs (0.85, 1.31, and 1.22 Å for H, T_I_, and T_II_ TS structures, respectively) are much larger than the corresponding glycosylation value (0.27 Å). Notice also that the hydrolysis value is significantly smaller than the transglycosylation ones, and of the same order as the values obtained for Glc hydrolysis by Osβ-gly (0.31–0.62 Å) (Badieyan et al., 2012; Wang et al., 2013). These results are in agreement with experimental observations indicating a more S_N_2 character for the glycosylation step than for the deglycosylation one (Kempton and Withers, 1992). Moreover, transglycosylation is predicted to have a transition state more advanced on the reaction coordinate than the hydrolysis one (RC values of −0.63, −0.17, and −0.20 Å for H, T_I_, and T_II_ TS structures, respectively).

In an attempt to determine the robustness of the geometrical differences found between the hydrolysis and transglycosylation TSs, the corresponding free energy profiles at the QM(SCC-DFTB)/MM level were obtained by umbrella sampling molecular dynamics simulations (Supplementary Figures S5, S6). Both reactions show very similar free energy barriers (21.10 and 21.93 kcal/mol for H and T, respectively), being the transglycosylation one slightly higher. At the corresponding TSs, the average C1_FUC_-O_Acc_, C1_FUC_-OE2_Glu338_ and H_Acc_-OE2_Glu164_ distances are 1.95 ± 0.15, 2.60 ± 0.18, and 1.52 ± 0.26 Å for hydrolysis, and 2.11 ± 0.05, 2.31 ± 0.08, and 1.32 ± 0.03 Å for transglycosylation. Thus, the dissociative character of the TSs is maintained, as well as the late proton transfer, although at this level of theory the hydrolysis TS would be more advanced on the reaction coordinate (RC_H_ = −0.87 Å) than the transglycosylation one (RC_T_ = −1.04 Å). However, it is important to note the significant fluctuations on the distances measured for the hydrolysis TS, which may be due to the flatness of the free energy profile at this zone and are indicative of a wide ensemble of available TS configurations with different reaction coordinate values. For the transglycosylation reaction, these fluctuations are much smaller. Interestingly, the transglycosylation QM(SCC-DFTB)/MM potential energy maximum is more advanced than the hydrolysis one (as with QM = PBE0/TZVP), which suggests that the apparent switch in TS localization along the reaction coordinate is a consequence of the introduction of dynamics. On the other hand, the more compact TS geometries when compared to the QM = PBE0/TZVP ones (sum of C1_FUC_-O_Acc_ and C1_FUC_-OE2_GLU338_ distances) is already present at the potential energy maxima, indicating that it is likely a consequence of the choice of QM level and not to the umbrella sampling simulations. QM(SCC-DFTB)/MM umbrella sampling simulations have been reported for hydrolysis and transglycosylation of Glc substrates catalyzed by a family GH3 β-glucosidase. Transglycosylation TS presented C1_GLC_-O_Acc_, C1_GLC_-O_Nuc_ and H_Acc_-O_Base_ distances of 3.0 ± 0.2, 1.9 ± 0.1, and 1.6 ± 0.1 Å, which are relatively similar to those obtained here at the QM = PBE0/TZVP level. However, the hydrolysis TS had the C1_GLC_-O_Acc_ bond practically formed (1.5 ± 0.04 Å) and the proton at a distance of 2.06 ± 0.08 Å from the catalytic base (although right before the TS this distance had shortened to 1.4–1.5 Å) (Geronimo et al., 2018). Therefore, water was predicted not to be activated by the basic residue, which is not what we observe in our calculations. The authors also related these geometric parameters to the lack of oxocarbenium ion-like character of this TS and a preference of the GH3 −1 subsite for a TS in a ^4^C_1_ conformation. (Geronimo et al., 2018). In the present system, the C1_FUC_-O5_FUC_ bond becomes shorter from reactants to the corresponding transition states accordingly to its partial double bond character along the nucleophilic attack. Hence, the positive charge on C1_FUC_ increases in between 0.24 and 0.27 au from reactants to the transition state structures (Supplementary Table S9 for QM(PBE0/TZVP)/MM and QM(large)/MM) while O5_FUC_ in the fucose ring becomes less negative (around 0.06 au) (with the QM(small)/MM partition the change on C1_FUC_ is slightly bigger (0.29–0.30 au), Supplementary Table S10). Those charge variations confirm the oxocarbenium ion-like character observed in deglycosylation reactions (hydrolysis as well as transglycosylation) in the GH1 β-glucosidases family. The ring puckering is very similar in both reactions, with Cremer-Pople ϕ values of 41° (for H), 44 and 47° (for T_I_ and T_II_) and ϕ angles of 224° (H), 224, and 221° (for T_I_ and T_II_), which correspond to a conformation between ^4^H_3_ and ^4^E. For the transglycosylation TS in family GH3 β-glucosidase mentioned above, a ^4^H_3_ conformation was predicted. (Geronimo et al., 2018).

It can be observed from the QM(PBE0/TZVP)/MM data in Supplementary Table S9 that there are only very small differences on the atomic charges of the atoms involved in the hydrolysis and transglycosylation reactions, in accordance with the small differences obtained in the energy barriers for the two kinds of deglycosylation processes. The lower energy barrier for hydrolysis might be due then to the more negative charge on the oxygen atom of the nucleophilic water in comparison to the negative charge value on the O4 atom of the glucose acceptor molecule.

**Figure 7:**
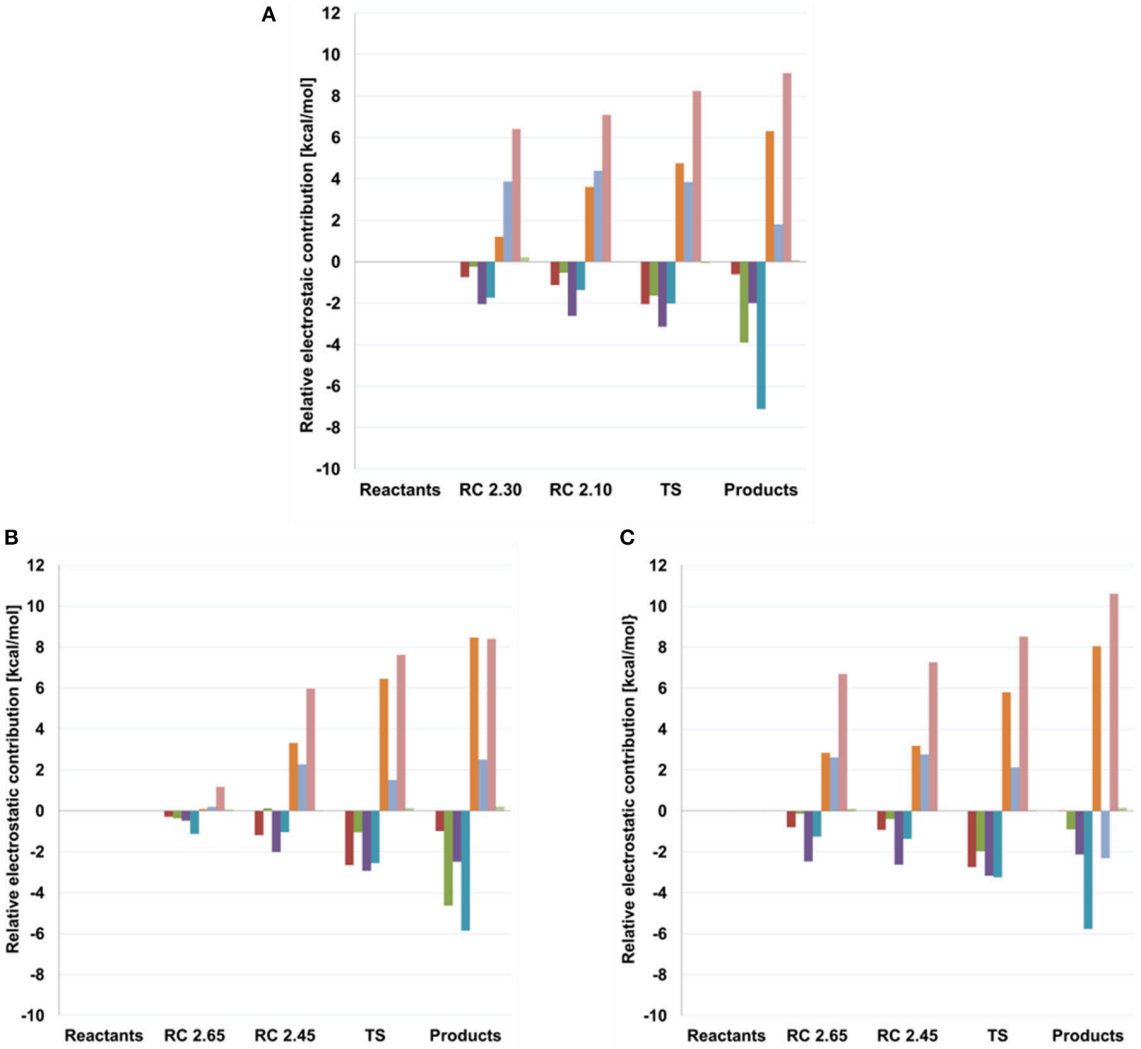
Electrostatic Interaction Analysis (in kcal/mol) of residues in the−1 subsite for **(A)** hydrolysis and both transglycosylation **(B)** T_I_ and **(C)** T_II_ frames studied in this work. Calculations were performed on the structures obtained with QM(small)/MM and at the QM = (PBE0/TZVP) level. Values, with respect to reactant, are given at different points along the reaction coordinate (RC), the transition states (TS) and products. Red: HIS 119, dark green: GLN 18, purple: ASN 163, dark blue: ASN 282, orange: TYR 284, light blue: GLU 392, pink: ARG 75, light green: CYS 164.

As can be seen in Figure 6 and Table 4, some interactions between the substrates or with the surrounding network of water molecules also vary along the two reactions studied. For transglycosylation, the hydrogen of the 3-OH group of the Glc acceptor substrate (adjacent to the attacking 4-OH_GLC_), in going from reactant to TS, approaches O5_FUC_ (by 0.53 and 0.38 Å for T_I_ and T_II_, respectively) and O4_FUC_ (by 0.12 and 0.51 Å for T_I_ and T_II_, respectively). In the hydrolysis reaction, a water molecule (WAT432) establishes a bridge between the oxygen atom of the attacking water (WAT431) and that of the 4-OH_FUC_ group. The O_WAT431_-H2_WAT432_ and the H1_WAT432_-O4_FUC_ distances show little variation from reactant to TS, but at the product the O_WAT431_-H2_WAT432_ distance increases by 0.91 Å due to a rotation of WAT432 (as O_WAT431_ has formed the new bond with C1_FUC_), which now points its H2_WAT432_ toward O5_FUC_ (*d*(H2_WAT432_-O5_FUC_) is 3.58 Å at reactants, 3.33 Å at TS and 1.95 Å at products). Thus, WAT432 and 3-OH_GLC_ seem to share a similar role in stabilizing the hydrolysis and transglycosylation TS, respectively, *via* interaction with the oxocarbenium ion. This kind of interactions have been identified in retaining glycosyltransferases to help the reaction, with the difference that due to the different disposition of the substrates they serve leaving group departure (Gómez et al., 2015). It is interesting to notice that the hydrogen atom of this 4-OH_FUC_ group is in turn interacting with Glu392; the H4O_FUC_-OE1_GLU392_ interaction becomes shorter along the hydrolysis and the T_I_ paths (it is not present along the T_II_ energy profile because a water molecule is located as a H-bonding bridge between O4_FUC_ and OE1_GLU392_). Moreover, the presence of Fuc instead of Glc as substrate (differing on the 4-hydroxyl configuration) has provoked a rotation of the Glu392 sidechain. The analysis of electrostatic contributions to the potential energy barrier of residues at the −1 subsite (Figure 7) show that Glu392 is stabilizing both the hydrolysis and transglycosylation TSs, being the effect larger for hydrolysis. In fact, this residue is the one showing the largest difference between the two competitive reactions, suggesting that altering this interaction could be a way to increase the T/H ratio. This observation could be related to the reported transglycosylation increase in the Asn390Ile and Phe401Ser mutants, both residues in contact with Glu392; Asn390 is H-bonding to Glu392 so that its mutation is likely to alter the Glu392-Fuc interaction. Three other residues interact with Fuc hydroxyl groups: In this case, an according to the electrostatic interactions analysis, they provide a small destabilization effect in this deglycosylation steps: Asn163, which interacts with the 2-OH_FUC_ group, with similar values for hydrolysis and transglycosylation; His119, that interacts with O3_FUC_ and O2_FUC_, and provides a slightly larger destabilization effect for transglycosylation; and Gln18, that interacts with O3_FUC_ with similar effect for both TSs.

In the hydrolysis and transglycosylation steps here studied, the nucleophile Glu338 presents strong interactions with the 2-OH_FUC_ group, Tyr284, Arg75, and Asn282. The H2O_FUC_-OE1_GLU338_ distance increases as the Glu338 residue moves away with the breakage of the C1_FUC_-OE2_GLU338_, although the H-bond interaction is maintained along the three reaction pathways and the negative charge of Glu338 also increases (around 0.3 au from reactants to the transition states, Supplementary Table S9). Concomitantly with those molecular changes, Tyr384 side-chain moves away from O5_FUC_ while it approaches OE2_GLU338_ atom (which, as mentioned, is increasing its negative charge). It has been suggested that the short hydrogen bond with the hydroxyl group of the equivalent tyrosine residue of a β-galactosidase could facilitate the elimination of the nucleophile protein residue (Bráa et al., 2010). The analyses depicted in Figure 7 show this increasing stabilization by Tyr284 as reaction proceeds (by 4.76 and 6.12 kcal/mol at the hydrolysis and transglycosylation TSs and 6.31 and 8.26 kcal/mol at their respective products). The electrostatic stabilization of the TSs as compared to the reactants provided by Arg75, that tends to slightly approach the OE1_GLU338_ as reaction proceeds, is the largest one (by 8.24 and 8.06 kcal/mol for hydrolysis and transglycosylation, respectively). These results qualitatively agree with the much lower activity measured when these two residues are mutated. Finally, Asn282 is predicted to have a moderate destabilizing effect, especially for the products. This residue interacts with both catalytic Glu338 and Glu164, thus the results indicate that its interaction with Glu164, which will go from a total −1 au charge to neutral, is the predominant one.

The residues that directly interact with the nucleophile (Tyr284, Asn282, Arg75) or with the acid/base catalyst (Asn163; which also interacts with O2_FUC_ and Arg75) are part of the ones identified to increase the T/H ratio when properly mutated (Teze et al., 2014) Bissaro et al. recently proposed that mutations impairing the optimized electron displacement system that the enzyme has for catalyzing hydrolysis (which includes the nucleophile and the oxocarbenium TS), results in the destabilization of the deglycosylation TSs and leads to accumulation of the glycosyl-enzyme intermediate; such accumulation has been linked to an increased T/H ratio (Bissaro et al., 2015a). Hydrophobic interactions (e.g., C-H – π interactions) augmenting the affinity of the enzyme for glycoside-like acceptors is another factor that can favor transglycosylation. (Tran et al., 2010; Bissaro et al., 2015a,b). In the present system the acceptor substrate can interact with Trp312. On the other hand, mutation of the catalytic acid/base Glu to Asp in NkBgl also catalyzed unforeseen transglycosylation (Jeng et al., 2012). The crystal structure of the mutant revealed a water molecule close to the shorter Asp chain and the anomeric carbon; thus, it was proposed that Asp would play its role through this nearby water molecule. A structurally conserved water molecule observed in a wild-type enzyme belonging to family GH101, was also proposed to enable a Grotthuss proton shuttle between the protein residue carboxylate and the glycosidic oxygen (Gregg et al., 2015). Still, the particular reasons why all these mutations in the −1 subsite favor transglycosylation over hydrolysis have not been fully unveiled.

The small differences in energy barriers these mutagenesis experiments imply and the high dimensionality of enzymatic systems, make it a great challenge to reproduce the effect of mutation computationally or to clearly identify its origin. For example, in the case of Tyr284 a transglycosylation yield of 76 % (but a lower rate) was measured for the Tyr284Phe mutant as compared to a 36 % for the WT enzyme (Teze et al., 2014). This was interpreted as a destabilizing effect produced by the mutation that affected more hydrolysis than transglycosylation. Our qualitative analysis of electrostatic interactions, though, estimates that in WT Ttβ-gly Tyr284 provides a slightly higher stabilization in the transglycosylation case. Attempts to reproduce the experimental observations by directly introducing the Tyr284Phe mutation on frames H, T_I_, and T_II_ and recalculating the potential energy profiles also failed. Thus, further computational studies of mutants taking into account structural rearrangements upon mutation (even if small) would be needed to try to reproduce these trends. However, and more importantly, these analyses are not able to capture the subtle changes in energy barriers involved in these cases, which are below the current “chemical accuracy.”

## Conclusions

Here we have presented a QM(DFT)/MM study of the glycosylation, hydrolysis and transglycosylation steps catalyzed by wild type *Thermus thermophilus* β-glycosidase (family GH1), a retaining glycosyl hydrolase for which a transglycosylation yield of 36 % has been determined experimentally for *p*NP-Fuc and BnON(Me)-Glc substrates.

In the glycosylation step (first step of the double-displacement mechanism of retaining GHs), the *p*NP-Fuc substrate establishes a strong network of hydrogen bonds at the−1 subsite and is found to be bound in an only slightly distorted ^4^C_1_ conformation, contrary to what has been reported when the substrate is a Glc disaccharide. At the transition state for this step, that has a strong oxocarbenium character (close to ^4^H_3_ conformation but with some ^4^E characteristics), proton transfer from Glu164 to the leaving group has not yet started but it takes place at a OE2_GLU338_-C1_FUC_ distance of ~2 Å with the QM(large)/MM partition and the PBE0/TZVP QM level. The potential energy barrier at this level of theory (20.4–22.0 kcal/mol) is in qualitative agreement with the experimental barrier (17.1 kcal/mol). The use of a smaller QM region increases this value by more than 5 kcal/mol. The size of the QM region also changes the location of the TS along the reaction coordinate. Thus, it seems to affect the nucleophilic and acid/base characteristics of Glu338 and Glu164, respectively.

For the hydrolysis and transglycosylation reactions (competing reactions in the deglycosylation step), a significant reduction of the barrier heights is also observed with the QM(large)/MM partition, although the T vs. H trends are well reproduced with the QM(small)/MM one provided that the PBE0/TZVP level is used to obtain the energies (or also for geometry optimization). The transglycosylation potential energy barrier is predicted to be 1.2–2.6 kcal/mol higher than the hydrolysis one. Hydrolysis and transglycosylation transition states are both very loose, with strong oxocarbenium-like character and the proton not yet transferred. Their ring conformation falls between ^4^H_3_ and ^4^E and they present very similar atomic charges, except for the more negative charge of the oxygen atom of the attacking water when compared to that of the attacking 4-OH group of Glc. Structural differences appear between the glycosylation and the deglycosylation steps, with the former having a more pronounced S_*N*_2 character. The proton transfer is also more advanced in the deglycosylation TSs that was the proton transfer in the glycosylation one. Differences between the hydrolysis and the transglycosylation TSs are also observed, but they are very dependent on the level of theory used. At the QM(PBE0/TZVP)/MM level, the potential energy maxima for transglycosylation is more advanced on the reaction coordinate than the hydrolysis one: has shorter O4_GLC_-C1_FUC_ (forming bond) distance and longer OE2_GLU338_-C1_FUC_ (breaking) distance, although the H_Acc_ proton is closer to the Glu164 base in hydrolysis. QM(SCC-DFTB)/MM free energy maxima show the inverted situation, although large geometric fluctuations are seen for the hydrolysis TS, indicating a large ensemble of TS configurations covering a range of reaction coordinate values. Interestingly, some interactions between the substrates (3-OH_GLC_ group) or with neighboring water molecules (WAT432) are identified that stabilize the oxocarbenium TSs through interaction with O5_FUC_ and O4_FUC_. An analysis of electrostatic interactions of the substrates with the residues at the−1 subsite has also been performed. However, correlation with experimental mutagenesis studies found to alter the T/H ratio is very challenging due to the small differences on energy barriers involved and potential structural (even if small) rearrangements upon mutation. The analysis suggests, though, that perturbing the Glu392-Fuc interaction could increase the T/H ratio, even by direct mutation of this residue or indirectly as reported experimentally in the N390I and F401S cases.

## Author Contributions

SR-T performed the calculations. SR-T, ÀG-L, and LM wrote the manuscript. SR-T, JL, ÀG-L, and LM contributed to the analysis and discussion of the results.

### Conflict of Interest Statement

The authors declare that the research was conducted in the absence of any commercial or financial relationships that could be construed as a potential conflict of interest.

## Footnotes

^1^The PyMOL Molecular Graphics System, Version 2.0 Schrödinger, LLC.
