# Supplementary material for: Comparing Hydrolysis and Transglycosylation Reactions Catalyzed by Thermus thermophilus β-Glycosidase. A Combined MD and QM/MM Study
Source: Front Chem. 2019 Apr 10;7:200. doi: 10.3389/fchem.2019.00200 (PMC6467970; doi:10.3389/fchem.2019.00200)

Comparing Hydrolysis and Transglycosylation Reactions Catalyzed by *Thermus thermophilus* β-glycosidase. A Combined MD and QM/MM Study

Sonia Romero-Téllez[1,2], José M. Lluch[1,2], Àngels González-Lafont[1,2,*], Laura Masgrau[1,2,*]

[1]Departament de Química, Universitat Autònoma de Barcelona, Cerdanyola del Vallès, Barcelona, Spain

[2]Institut de Biotecnologia i de Biomedicina, Universitat Autònoma de Barcelona, Cerdanyola del Vallès, Barcelona, Spain

Supplementary information

Supplementary Scheme S1. Atom type scheme of the *p*NP-Fuc substrate of the glycosylation step. Atom types from fucose moiety belong to GLYCAM06j force field and *p*NP atom types from gaff force field.

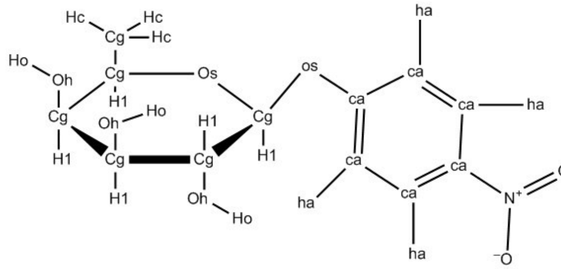

Supplementary Table S1. Frcmod file with missing parameters of the system.

BOND

os-Cg	285.0	1.460

ANGLE

os-Cg-H2	60.00	110.00
os-Cg-Os	100.00	112.00
ca-os-Cg	66.103	117.960

DIHE

Oh-Cg-Cg-os	1	1.010	0.000	-3
Oh-Cg-Cg-os	1	0.000	0.000	-2
Oh-Cg-Cg-os	1	0.020	180.000	1
ca-os-Cg-H2	3	1.150	0.000	3.000
ca-os-Cg-Os	3	1.150	0.000	3.000
ca-os-Cg-Cg	3	1.150	0.000	3.000
os-Cg-Cg-H1	1	0.05	0.0	3.

os-Cg-Cg-Cg	1	-0.27	0.0	1.
os-Cg-Os-Cg	1	0.96	0.0	-3.

Supplementary Table S2. Analysis over the MD simulation of the most populated hydrogen-bonds between *p*NP-Fuc acting as a donor and protein residues at the active site. H-bond occupancies are defined as the fraction of frames the bond is present. Only H-bond occupancies above 10% are included. The average donor to acceptor heavy atoms distances of the bonds when present are also given.

Acceptor	Donor	H-bond occupancy %	Average Distance (Å)
**$OE1_{GLN18}$**	$O3_{FUC}$	79.33	2.71
**$OE1_{GLU392}$**	$O4_{FUC}$	62.53	2.79
**$OE1_{GLU338}$**	$O2_{FUC}$	46.60	2.84
**$OE2_{GLU338}$**	$O2_{FUC}$	16.29	2.84
**$OE1_{GLN18}$**	$O4_{FUC}$	0.19	2.87

Supplementary Table S3. Analysis over the MD simulation of the most populated hydrogen-bonds between substrate atoms and protein residues at the active site. H-bond occupancies are defined as the fraction of frames the bond is present. Only H-bond occupancies above 10% are included. The average distance of the bonds when present between donor and acceptor heavy atoms are also given.

Substrate atom	Protein atom	H-bond occupancy %	Average Distance (Å)
**$O4D_{pNP428}$**	$OE2_{GLU164}$	90.61	2.76
**$O2_{FUC}$**	$ND2_{ASN163}$	46.81	2.89
**$O3_{FUC}$**	$OE2_{HIE119}$	34.44	2.86
**$O3_{FUC}$**	$OE1_{TRP393}$	16.6	2.91
**$H3O_{FUC}$**	$OE1_{TRP393}$	14.13	2.90
**$H2O_{FUC}$**	$ND2_{ASN163}$	3.96	2.93
**$H4O_{FUC}$**	$NE2_{GLN18}$	1.6	2.91
**$O2_{FUC}$**	$OE2_{GLU164}$	1.17	2.76

Supplementary Table S4. Analysis over the MD simulation of the most populated hydrogen-bonds between protein residues at the active site. H-bond occupancies are defined as the fraction of frames the bond is present. Only H-bond occupancies above 10% are included. The average distance of the bonds when present between donor and acceptor heavy atoms are also given.

**Acceptor**	**Donor**	**H-bond occupancy %**	**Average Distance (Å)**
$OE1_{GLU164}$	$ND2_{ASN282}$	92.53	2.81
$OE2_{GLU338}$	OH_{TYR284}	88.99	2.76

Supplementary Table S5. Analysis over the MD simulation of the most populated hydrogen-bonds between *p*NP-Fuc substrate and Solvent molecules. H-bond occupancies are defined as the fraction of frames the bond is present. Only H-bond occupancies above 10% are included. The average distance of the bonds when present between donor and acceptor heavy atoms are also given.

**Acceptor**	**Donor**	**H-bond occupancy %**	**Average Distance (Å)**
**$O01_{pNP428}$**	Solvent	99.64	2.80
**$O5_{FUC}$**	Solvent	24.00	2.85
**$O4_{FUC}$**	Solvent	20.44	2.84
**$O02_{pNP428}$**	Solvent	4.95	2.88
**Solvent**	$O4_{FUC}$	4.35	2.81
**$O4D_{pNP428}$**	Solvent	0.93	2.89
**$N01_{pNP428}$**	Solvent	0.07	2.97

Supplementary Table S6. Distances (in Å) between selected atoms involved in the glycosylation step (G), with the QM(large)/MM partition and at the PBE0/TZVP level for the QM description. Subscripts I and II stand for snapshot I and II, respectively.

	Reactant		TS		Product	
	G_I	G_{II}	G_I	G_{II}	G_I	G_{II}
$d(C1_{FUC}\text{-}OE2_{GLU338})$	3.33	3.41	2.61	2.67	1.50	1.50
$d(C1_{FUC}\text{-}O4D_{pNP})$	1.41	1.41	2.33	2.33	3.17	3.63
$d(C1_{FUC}\text{-}O5_{FUC})$	1.38	1.38	1.24	1.24	1.35	1.36
$d(H_{GLU164}\text{-}O4D_{pNP})$	1.99	2.02	1.82	1.88	0.98	0.97
$d(H_{GLU164}\text{-}OE2_{GLU164})$	0.96	0.95	0.97	0.96	1.64	1.67
$d(H_{TYR284}\text{-}O5_{FUC})$	4.08	4.05	2.80	2.82	2.22	2.21
$d(H_{TYR284}\text{-}OE2_{GLU338})$	1.81	1.83	1.97	1.87	2.29	2.31
$d/H_{ARG75}\text{-}OE1_{GLU338})$	2.02	1.96	2.00	2.08	2.16	2.24
$d(H2O_{FUC}\text{-}OE1_{GLU338})$	2.09	2.37	1.82	1.85	1.78	1.78
$d(H4O_{FUC}\text{-}O_{GLU392})$	1.96	1.89	2.22	2.23	2.30	2.42
$d(H_{ASN163}\text{-}O2_{FUC})$	2.04	2.21	2.00	2.12	1.97	2.06

Supplementary Table S7. Distances (in Å) between selected atoms involved in the glycosylation step (G), with the QM(small)/MM partition and at the PBE0/TZVP levels for the QM description. Subscripts I and II stand for snapshot I and II, respectively.

	Reactant		TS		Product	
	G_I	G_{II}	G_I	G_{II}	G_I	G_{II}
$d(C1_{FUC}\text{-}OE2_{GLU338})$	3.28	3.29	2.56	2.48	1.47	1.48
$d(C1_{FUC}\text{-}O4D_{pNP})$	1.40	1.40	2.31	1.76	3.52	3.66
$d(C1_{FUC}\text{-}O5_{FUC})$	1.39	1.40	1.24	1.32	1.37	1.37
$d(H_{GLU164}\text{-}O4D_{pNP})$	2.07	2.49	1.89	1.45	0.98	0.98
$d(H_{GLU164}\text{-}OE2_{GLU164})$	0.95	0.96	0.96	1.01	1.63	1.64
$d(H_{TYR284}\text{-}O5_{FUC})$	4.09	4.02	2.79	2.90	2.23	2.21
$d(H_{TYR284}\text{-}OE2_{GLU338})$	1.79	1.74	1.93	1.91	2.48	2.39
$d/H_{ARG75}\text{-}OE1_{GLU338})$	1.97	2.00	1.92	1.94	1.99	2.01
$d(H2O_{FUC}\text{-}OE1_{GLU338})$	1.89	1.96	1.73	1.74	1.76	1.77
$d(H4O_{FUC}\text{-}O_{GLU392})$	1.80	1.80	2.17	2.31	2.32	2.83
$d(H_{ASN163}\text{-}O2_{FUC})$	1.98	2.54	1.95	2.01	1.95	1.95

Supplementary Table S8. Distances (in Å) between selected atoms involved in each reaction step for the reactant, transition state (TS) and product of the hydrolysis (H) and transglycosylation (T_I and T_{II}, corresponding to the two different frames studied) steps. The results correspond to QM(small)/MM and at the PBE0/TZVP level. The Acc subscript refers to the acceptor water and glucose moieties in hydrolysis and transglycosylation processes, respectively.

	**Reactant**			**TS**			**Product**		
	**H**	**T_I**	**T_{II}**	**H**	**T_I**	**T_{II}**	**H**	**T_I**	**T_{II}**
***d*($C1_{FUC}$-$OE2_{GLU338}$)**	1.47	1.48	1.47	2.99	3.34	3.35	3.18	3.36	3.36
***d*($C1_{FUC}$-O_{Acc})**	3.67	3.38	3.38	2.25	2.15	2.17	1.39	1.41	1.42
***d*($C1_{FUC}$-$O5_{FUC}$)**	1.37	1.37	1.37	1.25	1.25	1.25	1.40	1.39	1.39
***d*(H_{Acc}-$OE2_{GLU164}$)**	1.78	1.76	1.71	1.45	1.44	1.40	0.96	0.96	0.96
***d*(H_{TYR284}-$O5_{FUC}$)**	2.11	2.24	2.21	2.93	3.13	3.17	3.83	4.04	4.09
***d*(H_{TYR284}-$OE2_{GLU338}$)**	2.50	2.49	2.45	1.89	1.85	1.85	1.77	1.76	1.76
***d*(H_{ARG75}-$OE1_{GLU338}$)**	1.97	2.00	1.98	1.99	2.10	1.98	1.99	2.06	1.92
***d*($H2O_{FUC}$-$OE1_{GLU338}$)**	1.76	1.77	1.74	1.77	1.77	1.68	1.90	1.98	1.86
***d*($H4O_{FUC}$-O_{GLU392})**	2.41	3.03	4.25	2.21	3.18	4.24	1.84	1.88	3.51
***d*(H_{ASN163}-$O2_{FUC}$)**	1.97	1.91	1.92	1.90	1.87	1.87	1.90	1.94	1.88
***d*($H3O_{GLC}$-$O4_{FUC}$)**	-	2.99	2.96	-	2.52	2.56	-	3.23	3.12
***d*($H3O_{GLC}$-$O5_{FUC}$)**	-	3.96	4.12	-	3.41	3.62	-	1.89	2.00
***d*($H3O_{GLC}$-$O4_{GLC}$)**	-	2.30	2.30	-	2.37	2.37	-	2.52	2.52
***d*($O3_{GLC}$-$H2_{WAT433}$)**	-	2.02	2.17	-	2.52	2.19	-	3.23	2.14
***d*(O_{WAT431}-$H2_{WAT432}$)**	1.76	-	-	1.82	-	-	2.66	-	-
***d*($H2_{WAT431}$-O_{WAT433})**	2.06	-	-	2.26	-	-	2.73	-	-
***d*($H2_{WAT432}$-$O5_{FUC}$)**	3.64	-	-	3.36	-	-	2.02	-	-
***d*($H1_{WAT432}$-$O4_{FUC}$)**	2.38	-	-	2.33	-	-	2.57	-	-

Supplementary Table S9. Selected NPA atom charges (in a.u) for the large QM region at QM(PBE0/TZVP) level. The Acc subscript refers to the acceptor water and glucose moieties in hydrolysis and transglycosylation processes, respectively.

	Reactant			TS			Product		
	**H**	**T_I**	**T_{II}**	**H**	**T_I**	**T_{II}**	**H**	**T_I**	**T_{II}**
**$OE1_{GLU164}$**	-0.89	-0.90	-0.89	-0.88	-0.88	-0.87	-0.74	-0.73	-0.73
**$OE2_{GLU164}$**	-0.84	-0.85	-0.85	-0.85	-0.86	-0.85	-0.74	-0.73	-0.74
**H_{TYR284}**	0.51	0.50	0.50	0.53	0.53	0.53	0.54	0.53	0.53
**$OE1_{GLU338}$**	-0.77	-0.77	-0.78	-0.90	-0.90	-0.90	-0.90	-0.89	-0.90
**$OE2_{GLU338}$**	-0.63	-0.63	-0.62	-0.88	-0.87	-0.87	-0.88	-0.88	-0.88
**$C1_{FUC}$**	0.45	0.46	0.45	0.72	0.70	0.70	0.42	0.44	0.45
**$H1_{FUC}$**	0.20	0.20	0.21	0.22	0.22	0.23	0.15	0.15	0.15
**$O5_{FUC}$**	-0.57	-0.57	-0.57	-0.51	-0.52	-0.51	-0.61	-0.62	-0.60
**$H2O_{FUC}$**	0.50	0.50	0.51	0.53	0.53	0.54	0.52	0.52	0.53
**O_{ACC}**	-1.04	-0.79	-0.80	-1.05	-0.81	-0.82	-0.77	-0.69	-0.64
**H_{ACC}**	0.53	0.53	0.53	0.54	0.55	0.54	0.54	0.55	0.54

Supplementary Table S10. Selected NPA atom charges (in a.u) for the small QM region at QM(PBE0/TZVP) level. The Acc subscript refers to the acceptor water and glucose moieties in hydrolysis and transglycosylation processes, respectively.

	Reactant			TS			Product		
	**H**	**T_I**	**T_{II}**	**H**	**T_I**	**T_{II}**	**H**	**T_I**	**T_{II}**
**$OE1_{GLU164}$**	-0.91	-0.92	-0.91	-0.89	-0.88	-0.88	-0.75	-0.74	-0.74
**$OE2_{GLU164}$**	-0.85	-0.86	-0.86	-0.85	-0.86	-0.86	-0.74	-0.74	-0.74
**H_{TYR284}**	0.51	0.50	0.50	0.54	0.54	0.53	0.54	0.54	0.54
**$OE1_{GLU338}$**	-0.74	-0.75	-0.76	-0.89	-0.89	-0.90	-0.89	-0.88	-0.89
**$OE2_{GLU338}$**	-0.62	-0.63	-0.62	-0.91	-0.90	-0.90	-0.90	-0.91	-0.90
**$C1_{FUC}$**	0.43	0.44	0.43	0.73	0.71	0.71	0.41	0.43	0.44
**$H1_{FUC}$**	0.21	0.21	0.22	0.24	0.23	0.24	0.16	0.16	0.16
**$O5_{FUC}$**	-0.57	-0.57	-0.56	-0.51	-0.51	-0.50	-0.61	-0.62	-0.61
**$H2O_{FUC}$**	0.51	0.51	0.51	0.54	0.54	0.54	0.53	0.53	0.53
**O_{ACC}**	-1.04	-0.80	-0.80	-1.06	-0.82	-0.83	-0.76	-0.61	-0.61
**H_{ACC}**	0.53	0.53	0.53	0.54	0.54	0.54	0.54	0.54	0.53

Supplementary Figure S1. RMSD analysis of **(A)** the protein backbone over 100 ns, excluding the first 4 residues and the last one, and **(B)** the heavy atoms of the *p*NP-Fuc substrate.

(A)

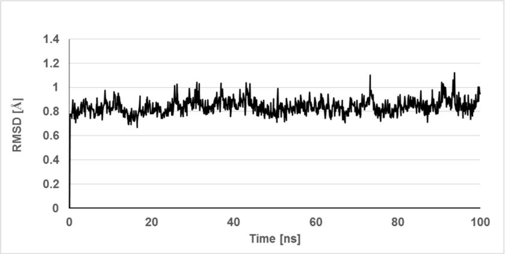

(B)

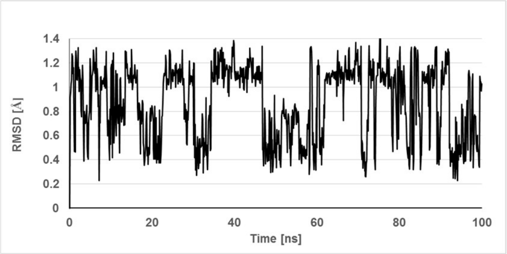

Supplementary Figure S2. Evolution of $C1_{FUC}$ - $O4D_{pNP}$ (in blue), $C1_{FUC}$ - $OE2_{GLU338}$ (in orange), H_{GLU164} - $O4D_{pNP}$ (in purple) and H_{GLU164} - $OE2_{GLU164}$ (in green) distances along the glycosylation reaction coordinate of G_I with the QM(small)/MM partition at the **(A)** PBE0/SVP and **(B)** PBE0/TZVP levels for the QM description. Distances are in Å.

(A)

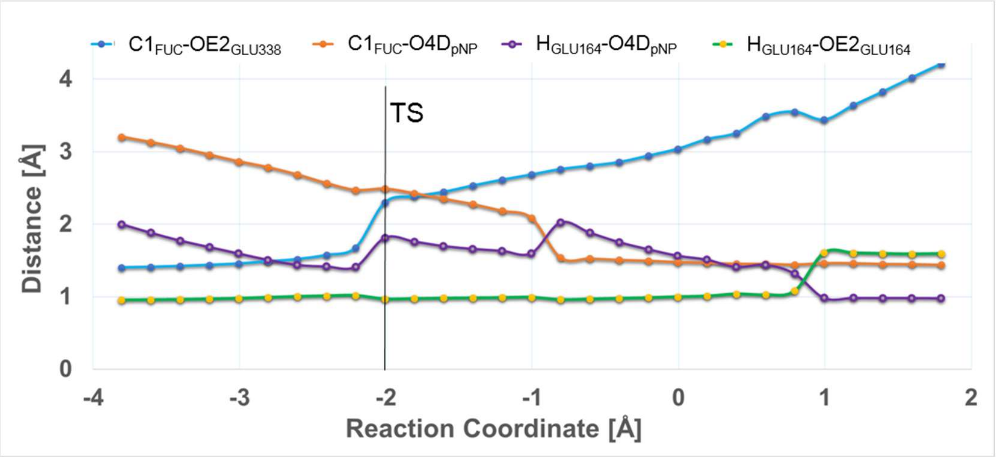

(B)

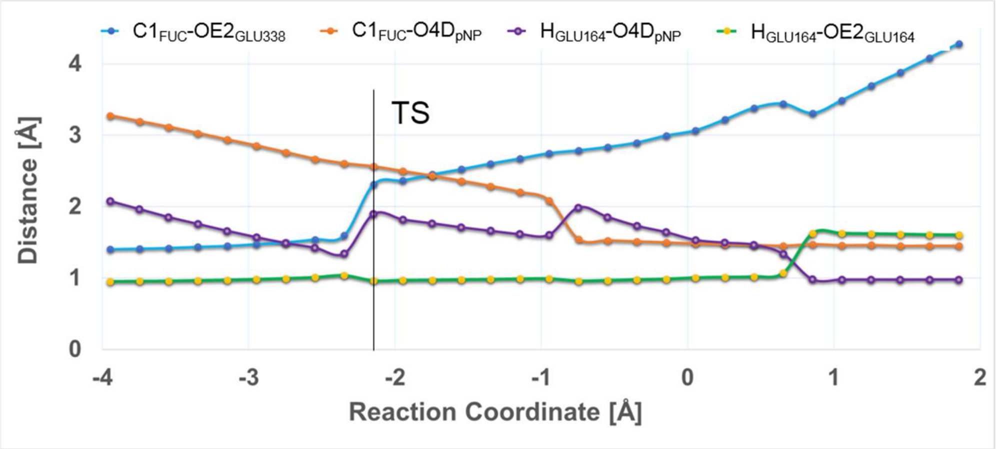

Supplementary Figure S3. Potential energy profile (in kcal/mol) for the glycosylation step, starting from an optimized, distorted conformation of the fucose ring of *p*NP-Fuc substrate. Calculations were performed with QM(PBE0/TZVP)/MM using the large QM region.

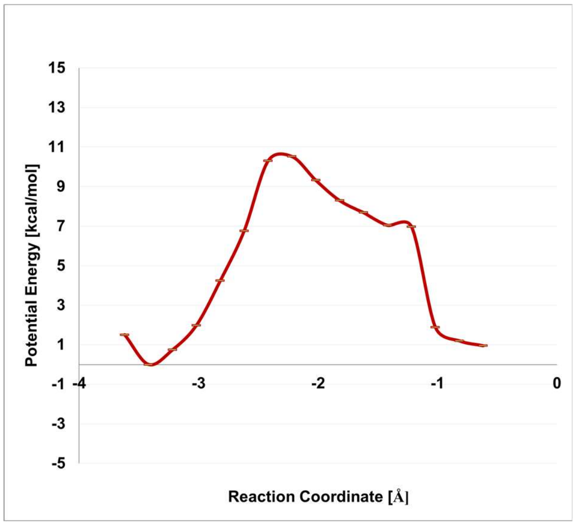

Supplementary Figure S4. Structural comparison of transglycosylation substrate **(A)** before and **(B)** after performing the refinement protocol involving QM(SCC-DFTB)/MM molecular dynamics described in the Results section for transglycosylation.

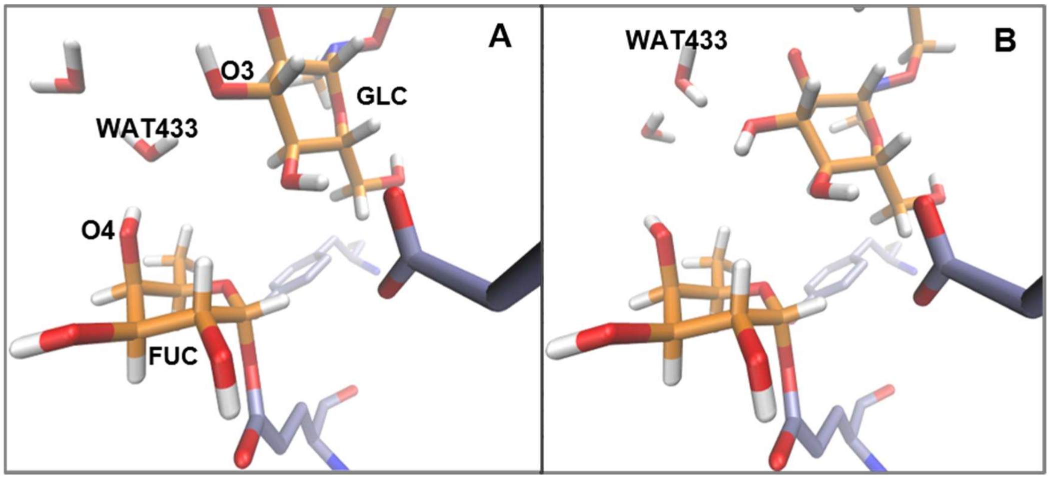

Supplementary Figure S5. Free energy profile (in kcal/mol) for the hydrolysis step (blue) and transglycosylation step (red). Calculations were performed by umbrella sampling calculations at the QM(SCC-DFTB)/MM level using the catalytic residues, substrates and one water (in Hydrolysis) as QM region.

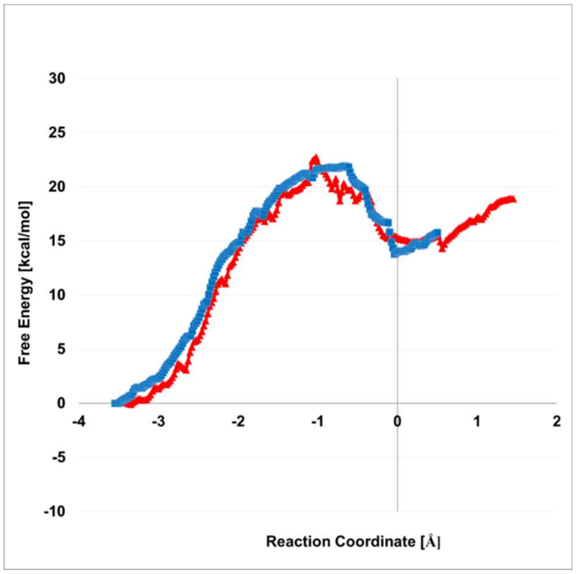

Supplementary Figure S6. Histogram from the umbrella sampling simulations of the (A) hydrolysis and (B) transglycosylation steps.

(A)

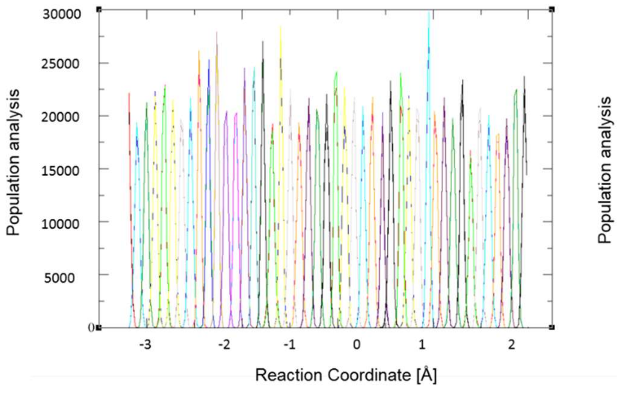

(B)

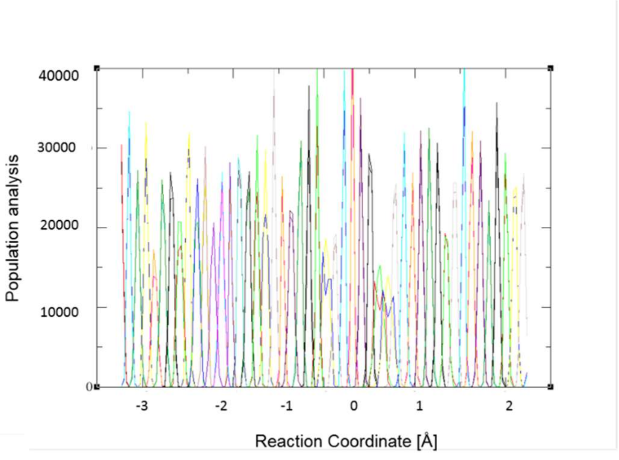

Supplement: Supplementary file 1 [file Data_Sheet_1.pdf]
